# *Clock*-Related Genes Mark a Developmental Cortical Maturation Program Associated with Stage-Resolved Responses to Prenatal Immune Activation

**DOI:** 10.3390/genes17091107

**Published:** 2026-09-12

**Authors:** Yilin Wang, Shanshan Li, Xin Jin

**Affiliations:** 1 Department of Applied Mathematics and Statistics, Whiting School of Engineering, Johns Hopkins University, Baltimore, MD 21218, USA; 2 School of Medicine, Nankai University, Tianjin 300071, China

**Keywords:** *Clock*-related genes, cortical development, synaptic maturation, BrainSpan-derived developmental program, prenatal immune activation, autism spectrum disorder, single-cell transcriptomics, weighted gene co-expression network analysis

## Abstract

**Background/Objectives:** Sleep and circadian disturbances are common in neurodevelopmental conditions, yet the developmental cortical programs linking clock-related transcriptional regulators to disease vulnerability remain unclear. **Methods:** Here, we integrated human developmental brain transcriptomes, weighted gene co-expression network analysis (WGCNA), human and mouse cortical single-cell atlases, prenatal immune activation transcriptomes, and ASD postmortem brain datasets to characterize the developmental architecture of BrainSpan-derived cortical programs and examine their behavior in perturbational and disease contexts. **Results:** In the BrainSpan frontal cortex, canonical clock-related genes followed structured but heterogeneous developmental trajectories rather than behaving as a coordinated oscillator-like unit. WGCNA identified a postnatal-rising BrainSpan-derived primary developmental module that was strongly associated with developmental age and enriched for synaptic signaling, neurotransmitter transport, ion transport, membrane excitability, cellular respiration, metabolic regulation, and proteostatic processes. Network analysis placed multiple canonical clock-related and clock-regulatory genes, including NPAS2, BHLHE40, BHLHE41, PER family members, RORA, NR1D1/2, and CLOCK, within a broader neuronal and homeostatic co-expression architecture, although their module-membership strengths varied substantially. Projection onto a human cortical developmental single-cell atlas revealed a non-uniform distribution of the corrected BrainSpan-derived developmental signature, with relatively higher scores in excitatory and inhibitory neuronal populations and lower scores in neuroblast and radial glial populations. A mouse cortical developmental single-cell atlas provided a comparative view of the stage- and cell-type-dependent expression of clock-related genes and the transferred developmental signature during corticogenesis. In a Poly(I:C)-based maternal immune activation dataset, litter-aware reanalysis identified stage-resolved genome-wide transcriptional responses following E12.5 exposure. However, neither the aggregate core clock-gene expression score nor the independently transferred BrainSpan-derived developmental signature showed a significant overall treatment effect or collection-stage-by-treatment interaction, indicating that this bulk dataset provides a perturbational context rather than evidence for selective disruption of the developmental program. An exploratory region-stratified analysis of GSE28521 yielded near-null effects for the BrainSpan-derived developmental signature, with confidence intervals crossing zero across all examined regions. These ASD postmortem findings were therefore treated as a boundary assessment rather than evidence of ASD-specific convergence. **Conclusions:** Collectively, these findings position clock-related genes as components of a developmentally regulated cortical maturation program enriched for neuronal signaling, synaptic maturation, metabolic regulation, and stress-response processes.

## 1. Introduction

Neurodevelopmental disorders are increasingly understood as disorders of early brain development in which genetic and environmental risks converge on cortical cell specification, neuronal differentiation, synaptic maturation, excitatory–inhibitory balance, glial regulation, and immune-homeostatic signaling. Large-scale genomic and transcriptomic studies have shown that neurodevelopmental disorder risk genes are enriched in developmental brain programs and converge on cortical neuronal and synaptic pathways, supporting a model in which vulnerability emerges from disrupted maturation of cortical circuits rather than isolated molecular defects [1,2]. Cell-type-resolved analyses have further revealed altered neuronal activity, neuroinflammatory signatures, and glial-associated molecular subtypes in the cortex, indicating that both neuronal and non-neuronal compartments contribute to disease-relevant cortical dysregulation [3,4,5,6]. In parallel, clinical studies consistently report a high burden of sleep and circadian disturbances in individuals with ASD, including insomnia, altered sleep–wake timing, and circadian rhythm sleep–wake problems, which are associated with behavioral and cognitive difficulties [6,7,8,9]. These observations raise an important question: are clock-related molecular programs merely downstream correlates of sleep disturbance, or do they participate more directly in cortical developmental vulnerability?

Canonical circadian clock genes have traditionally been studied as components of interlocking transcriptional–translational feedback loops that generate approximately 24-h rhythms. These loops are centered on core regulators such as *ARNTL*, *CLOCK*, *NPAS2*, *PER1*, *PER2*, *PER3*, *CRY1*, *CRY2*, *NR1D1*, *NR1D2*, *RORA*, *RORB*, *RORC*, *CSNK1D*, and *CSNK1E*, together with related clock-regulatory factors [10]. Accumulating evidence, however, indicates that clock-related genes are not restricted to systemic timekeeping or sleep regulation. In the central nervous system, molecular clock components are expressed in multiple neuronal and glial populations and can modulate neuronal activity, astrocytic and microglial function, cortical excitability, and synaptic plasticity with spatial and temporal specificity. Beyond their canonical rhythmic roles, clock-related transcriptional regulators intersect with metabolic regulation, mitochondrial oxidative metabolism, NAD+ cycling, redox homeostasis, stress adaptation, and immune regulation [11,12,13,14,15,16,17,18,19]. These functions are highly relevant to the developing cortex, where neuronal maturation requires coordinated control of synaptic activity, ion transport, mitochondrial support, proteostasis, and homeostatic stress responses. Clock-related genes may therefore function not only as components of circadian oscillators but also as broader transcriptional regulators embedded within cortical developmental programs.

Despite growing interest in circadian dysfunction in ASD and other neurodevelopmental disorders, three major gaps remain. First, much of the existing literature has focused on clinical sleep phenotypes, adult brain function, genetic association, or individual clock genes such as *PER2*, rather than on the developmental organization of clock-related programs across human cortical maturation [20,21,22,23,24,25]. Second, bulk postmortem studies cannot resolve whether clock-related developmental signals arise from neuronal maturation, glial states, vascular compartments, or changing tissue composition. Third, most studies have not determined whether clock-related genes operate as isolated circadian components or are integrated into broader co-expression networks linked to synaptic and homeostatic maturation. The BrainSpan developmental transcriptome provides a foundational resource for modeling age-resolved human brain gene expression, and previous co-expression studies have shown that developmental brain networks can illuminate mechanisms relevant to neurodevelopmental disorders [26,27,28]. More recently, single-cell and single-nucleus atlases of prenatal and postnatal human cortical development have enabled cell-type- and stage-resolved mapping of developmental gene programs across radial glia, excitatory neurons, inhibitory neurons, glial lineages, and vascular-associated populations [29,30]. Together, these resources enable the developmental trajectory, network architecture, and cellular localization of clock-related cortical programs to be examined at substantially higher resolution.

A further unresolved question is whether such clock-related developmental programs are sensitive to prenatal environmental perturbation. Maternal immune activation (MIA) is a well-established risk-relevant framework for studying how prenatal inflammatory challenge alters fetal brain development and increases susceptibility to neurodevelopmental disorders, including ASD- and schizophrenia-related phenotypes [31,32]. Poly(I:C)-based MIA models have been widely used to induce antiviral-like maternal immune responses and to investigate downstream effects on offspring cortical development, immune signaling, synaptic maturation, and behavior [33,34]. Importantly, the effects of MIA are not uniform but vary with gestational timing, developmental stage, brain region, cytokine response, and susceptible cell type [35,36,37,38]. These observations suggest that the transcriptional consequences of prenatal immune challenge may evolve across subsequent stages of cortical development. However, how clock-related developmental expression programs behave across corticogenesis after prenatal immune activation remains unclear.

Here, we integrated human BrainSpan frontal cortical transcriptomes, WGCNA, human and mouse developmental single-cell atlases, prenatal immune activation transcriptomes, and ASD postmortem brain datasets to define the developmental architecture of a BrainSpan-derived cortical developmental program and assess its behavior across external biological contexts. We identified a postnatal-rising BrainSpan-derived primary developmental module enriched for synaptic signaling, ion transport, neuronal activity, metabolic support, and proteostatic processes. Single-cell projection further indicated preferential representation of the signature in differentiated excitatory and inhibitory neuronal populations rather than early progenitor populations. We then used a Poly(I:C)-based maternal immune activation dataset as an external perturbational context to determine how cortical transcriptional responses evolve after a single prenatal immune challenge. Litter-aware analyses distinguished genome-wide stage-resolved responses from effects on the independently defined BrainSpan-derived developmental signature. GSE28521 was retained only as an exploratory disease-context boundary assessment and was not used as evidence for ASD-specific convergence of the BrainSpan-derived developmental program. Together, these analyses define clock-related genes as components of a developmentally regulated cortical co-expression program while placing prenatal immune activation and ASD postmortem data within appropriately bounded perturbational and disease-context frameworks.

## 2. Materials and Methods

### 2.1. Study Design

This study was designed as an integrative transcriptomic and single-cell analysis to define the developmental organization of clock-related genes and associated cortical programs and to evaluate their behavior in neurodevelopmental perturbation and disease contexts. The analytical workflow comprised six sequential components: (1) age-resolved modeling of canonical clock-related genes in the human frontal cortex; (2) weighted gene co-expression network analysis to identify a BrainSpan-derived primary developmental module; (3) projection of bulk-derived modules onto a human cortical developmental single-cell atlas; (4) cross-species evaluation using a mouse cortical developmental single-cell atlas; (5) analysis of prenatal immune activation using a maternal immune activation bulk transcriptomic dataset; and (6) region-stratified assessment of related module signatures in ASD postmortem brain tissue. BrainSpan was selected as the primary discovery cohort because it provides age-resolved human cortical gene-expression profiles spanning prenatal development to adulthood. The human developmental single-cell atlas was used to determine the cellular localization of BrainSpan-derived transcriptional programs and to distinguish broadly distributed clock-gene expression from cell-state-associated maturation signatures. GSE153162 was used to assess the developmental and cellular distribution of corresponding signatures during mouse corticogenesis. GSE166376 was selected as a perturbational-context dataset to characterize stage-resolved cortical transcriptional responses after a single E12.5 maternal immune challenge and to test whether the independently defined BrainSpan-derived developmental signature showed detectable treatment-associated changes. GSE28521 was retained as an exploratory region-stratified boundary assessment of projected developmental and clock-related signatures in heterogeneous human postmortem tissue; it was not used to validate the BrainSpan-derived developmental program or to establish ASD-specific molecular convergence. All analyses were conducted using R (version 4.5.0)- and Python 3-based bioinformatics workflows.

### 2.2. Human BrainSpan Frontal Cortex Dataset Processing

Transcriptomic data and sample metadata from the BrainSpan Atlas of the Developing Human Brain were retrieved and curated for frontal cortical regions. Samples were retained if they originated from frontal cortical structures and had available annotations for developmental age, sex, and cortical structure. Included regions comprised the dorsolateral, medial, orbital, and ventrolateral frontal cortex. Developmental age was harmonized as post-conception days and transformed as log2(post-conception days + 1) for downstream modeling. Samples were further classified into broad developmental periods: prenatal, birth–1 year, 1–2 years, childhood, adolescence, and adulthood. Gene-expression values were processed at the gene level, and genes with insufficient expression across retained samples were excluded before downstream modeling. Expression values were log-transformed where required and mapped to standardized gene symbols. For prenatal-versus-postnatal analyses, samples were categorized according to whether post-conception age occurred before or after birth. Cortical structure and sex were retained as covariates in regression-based analyses to reduce confounding by anatomical and demographic differences.

### 2.3. Definition of the Primary Developmental Module and Secondary Biological Reference Sets

The canonical clock-related gene set was predefined as 20 genes: ARNTL, CLOCK, NPAS2, PER1, PER2, PER3, CRY1, CRY2, NR1D1, NR1D2, RORA, RORB, RORC, BHLHE40, BHLHE41, CSNK1D, CSNK1E, TIMELESS, FBXL3, and FBXL21. This predefined set was used consistently for all primary enrichment analyses; no alternative reduced clock-gene subset was used to define statistical significance. Mouse orthologs of these genes were used for analyses involving murine datasets.

To distinguish the primary analytical object from secondary biological annotations, we defined one data-driven primary developmental module and several independent reference gene sets. The primary developmental module was identified from BrainSpan cortical transcriptomes using weighted gene co-expression network analysis (WGCNA). Module identification was performed independently of curated biological gene sets and was based exclusively on developmental expression patterns, including associations with continuous age and prenatal-to-postnatal transition. The selected BrainSpan-derived module was subsequently treated as the primary analytical program throughout the study.

Predefined biological gene sets were used only for downstream characterization and comparative analyses of the primary module. These secondary reference sets comprised clock-output, metabolic-support, neuronal activity/synaptic-maturation, proteostasis/stress-response, and immune-response gene sets. The categories were analyzed separately because they represent distinct biological processes rather than interchangeable components of a single “homeostatic” pathway. Representative genes included *TEF*, *HLF*, and *DBP* (clock-output regulation); *PPARGC1A* and *NAMPT* (metabolic regulation); *CAMK2A*, *EGR1*, *EGR2*, *EGR3*, *ARC*, *MEF2D*, *JUNB*, *FOS*, *FOSB*, *NPAS4*, *DLG4*, *SYT1*, and *HOMER1* (neuronal activity and synaptic maturation); *EIF4E* (translation-related regulation); and *UBE3A* (neurodevelopmental regulation).

For downstream analyses, the BrainSpan-derived primary developmental module was transferred as a fixed gene signature while retaining its original gene membership and module-connectivity information. Secondary reference gene sets, by contrast, were evaluated independently and were not interpreted as alternative representations of the primary module. In each external dataset, only genes overlapping the processed expression matrix were included in score calculation or enrichment analyses.

### 2.4. Developmental Trajectory Modeling of Clock-Related Genes

To assess age-dependent expression trajectories of core clock-related genes in the human frontal cortex, gene-wise generalized additive models (GAMs) were fitted using the mgcv framework. For gene g in sample i, Y_gi_, the response variable was log2-transformed gene expression [log2(RPKM + 1)], and developmental age was represented as age_log2 = log2(post-conception days + 1). The full model was specified as:

$$Y_{gi}=\beta_{0}+s\left(log_{2}\left(PCD_{i}+1\right),k=4\right)+\beta_{1}Sex_{i}+\beta_{2}Structure_{i}+s\left(Donor_{i},bs=“re”\right)+\epsilon_{gi}$$

The developmental-age effect was modeled using the default thin-plate regression spline basis in mgcv, with a maximum basis dimension of k = 4; donor identity was included as a random-effect smooth (bs = “re”) when repeated samples from the same donor were available. Models were fitted by restricted maximum likelihood (REML), with sex and cortical structure included as fixed-effect covariates. The significance of the developmental-age effect was assessed for each gene and adjusted for multiple testing using the Benjamini–Hochberg false-discovery-rate procedure. Fitted expression trajectories were visualized across developmental age, with individual samples colored by prenatal or postnatal status and birth marked as a reference point. Predicted expression values from the GAMs were calculated across an ordered developmental-age grid and row-scaled to generate heatmaps of developmental trajectory classes. Genes were classified descriptively as showing postnatal-rising, prenatal-high, or nonlinear/biphasic trajectories. These models were designed to characterize developmental changes in transcript abundance rather than circadian rhythmicity; phase, amplitude, period, and oscillator function were not inferred because the source datasets lacked time-of-day-controlled serial sampling.

### 2.5. Weighted Gene Co-Expression Network Analysis

Weighted gene co-expression network analysis was performed on BrainSpan frontal cortical transcriptomes to identify co-expression modules associated with cortical development. Before network construction, genes with low expression or low variance across samples were removed. The filtered expression matrix was used to construct a signed weighted co-expression network. A soft-thresholding power was selected to approximate scale-free topology while maintaining adequate network connectivity. Adjacency matrices were transformed into topological overlap matrices, and modules were identified by hierarchical clustering followed by dynamic tree cutting. Module eigengenes were calculated as the first principal component of each module.

Because the sign of a principal-component score is arbitrary, module eigengenes were oriented consistently for interpretation. For the primary developmental module, the eigengene was oriented so that its correlation with continuous developmental age was positive; when the initially returned eigengene showed the opposite orientation, both the eigengene and the corresponding signed module-membership coefficients were multiplied by −1. This convention affects only the displayed direction and does not alter module membership, correlation magnitude, statistical significance, or network topology.

Associations between module eigengenes and developmental traits were assessed by correlation analysis. Traits included continuous developmental age and prenatal-versus-postnatal status, and *p* values were adjusted across module–trait tests using the Benjamini–Hochberg procedure. For all revised downstream analyses, the postnatal-rising turquoise module was fixed as the BrainSpan-derived primary developmental module because it showed the strongest positive association with continuous developmental age and postnatal status. Biological annotations, including canonical clock-related, neuronal, metabolic, and other functional gene sets, were evaluated only after the primary developmental module had been defined and were not used to establish its developmental identity.

To evaluate the robustness of module-membership estimates to major measured covariates, we performed a covariate-adjusted sensitivity analysis while keeping the original turquoise-module membership fixed. For each gene in the primary developmental module, expression was residualized against developmental age, donor identity, sex, and cortical structure using the available BrainSpan metadata. Module-membership coefficients were then recalculated from the residualized expression matrix and compared with the original WGCNA-derived values. Changes in hub ranking were summarized by overlap among the top 50, 100, and 200 genes ranked by absolute module membership before and after residualization. This analyses were intended to assess internal concordance of gene-membership and hub rankings under covariate adjustment; they did not reconstruct a genome-wide residual co-expression network and therefore were not interpreted as a formal test of network or module preservation.

To quantify the distribution of canonical clock-related genes across the complete WGCNA landscape, enrichment was tested independently for each module using the predefined 20-gene canonical clock-related set described above. Of these 20 genes, 18 were represented in the WGCNA gene universe and were included in the enrichment analysis. One-sided Fisher’s exact tests were performed using all genes entering the WGCNA network as the background universe, and *p* values were corrected across modules using the Benjamini–Hochberg procedure. Module size, the number of represented canonical clock-related genes, odds ratios, nominal *p* values, and FDR-adjusted *p* values are reported in Supplementary Table S6. No alternative reduced clock-gene subset was used to define statistical significance in the revised analysis.

### 2.6. Module Eigengene Trajectory and Prenatal–Postnatal Comparison

The eigengene of the selected BrainSpan-derived primary developmental module was modeled across developmental age using a generalized additive model with log2-transformed post-conception days as a smooth term and sex and cortical structure as covariates. In parallel, module eigengene values were compared between prenatal and postnatal samples using a Wilcoxon rank-sum test and covariate-adjusted linear modeling. The postnatal–prenatal mean difference was calculated as the mean oriented module eigengene value in postnatal samples minus that in prenatal samples. Module eigengene values are first-principal-component scores on the native WGCNA eigengene scale; accordingly, the reported difference is expressed in eigengene-score units and should not be interpreted as a gene-expression fold change or standardized effect size. Spearman correlation was used to quantify the association between module eigengene values and continuous developmental age.

### 2.7. Functional Enrichment Analysis of Module Genes

Functional enrichment analysis was performed using genes assigned to the selected WGCNA module. Gene Ontology biological-process enrichment was conducted using an over-representation framework, and enrichment *p* values were adjusted using the Benjamini–Hochberg false-discovery-rate method. Enriched terms were manually grouped into major biological categories, including synaptic signaling, neurotransmitter transport and secretion, transmembrane and monatomic ion transport, regulation of membrane potential, protein localization and proteostasis, lysosomal organization, cellular respiration, and metabolic processes. Representative terms were visualized as −log10(FDR) values. To assess potential bias introduced by predefined gene-set selection, a sensitivity analysis was performed after removing all module genes overlapping manually curated clock-related gene sets. Functional enrichment was then repeated using the remaining module genes, and the resulting biological processes were compared with those obtained from the complete module.

### 2.8. Hub-Gene Identification and Module Architecture

Genes within the BrainSpan-derived primary developmental module were ranked by module membership (kME), defined as the correlation between each gene’s expression and the corresponding module eigengene. Module assignments and kME values for all canonical clock-related genes were also reported irrespective of whether they belonged to the primary module. *Clock-related*, clock-output, metabolic, neuronal maturation, and activity-responsive genes were treated as biological annotations rather than criteria defining module identity. Representative high-membership genes were visualized in an age-ordered heatmap using row-scaled expression values, with samples annotated by developmental period, age bin, cortical structure, and module eigengene value. Complete module assignments and module-membership statistics for canonical clock-related genes are provided in Supplementary Table S6.

### 2.9. Human Cortical Developmental Single-Cell Atlas Analysis

The human cortical developmental single-cell reference was obtained from the Brain Transcriptome Single-cell (BTS) Atlas reported by Kim et al. (2024) [39]. Processed atlas files were downloaded from Zenodo record 14177002, version v1.0.1, including the AnnData and Seurat objects of the BTS atlas. Metadata, major cell-type annotations, developmental-stage annotations, and UMAP coordinates provided by the original resource were used for downstream projection and visualization. Cells with available major cell-type and developmental-stage annotations were retained. Major cortical populations included excitatory neurons, inhibitory neurons, oligodendrocyte-lineage cells, neuroblasts, astrocytes, radial glia, oligodendrocyte precursor cells, microglia, endothelial cells, and other annotated minor populations. The single-cell analysis was designed as a descriptive localization analysis to map the expression distribution of the fixed BrainSpan-derived developmental signature across cortical cell states; it was not intended to reconstruct or validate the BrainSpan co-expression network within individual cell types. Module scores were calculated independently for four predefined gene programs: the BrainSpan-derived primary developmental module, canonical core clock gene set, clock-output gene set, and neuronal maturation gene set. For each gene set, only genes detected in the BTS expression matrix were used. Cell-level scores were projected onto the BTS UMAP embedding, summarized across major cell classes, and averaged within developmental-stage-by-cell-subtype groups to evaluate stage- and cell-state-specific organization of cortical clock-related developmental programs. Cell-level scores were therefore interpreted as descriptive signature-expression measures rather than independent biological replicates or evidence of within-cell-type network preservation. The numbers of donors and retained cells for the analyzed cell populations and developmental stages are summarized in Supplementary Table S7. The processed BTS atlas was used as supplied by the original study. No de novo cell-level quality-control filtering, doublet detection, batch integration, dimensionality reduction, clustering, UMAP construction, or cell-type annotation was performed for the present analysis. The published expression matrix, donor/sample metadata, developmental-stage annotations, major and subtype cell annotations, and UMAP coordinates were retained for downstream signature projection and descriptive visualization. Thus, quality-control and integration parameters from the original BTS atlas were not re-estimated or modified in the present study.

### 2.10. Single-Cell Visualization and Cell-Type Comparison of Module Scores

Cell-level module scores were projected onto UMAP embeddings to assess their spatial distribution across the cortical developmental landscape. Dot plots were used to visualize representative clock-related, clock-output, metabolic-support, neuronal maturation/activity-associated, and hub genes across major cell types. Dot size represented the fraction of cells expressing a gene within a cell type, whereas color represented scaled mean expression. Score distributions across major cell classes were summarized using boxplots. Mean primary developmental module scores were calculated for each available developmental-stage-by-cell-subtype combination. For visualization in the stage-by-subtype heatmap, subtypes represented by at least 300 cells across the full analyzed atlas were eligible for inclusion. To maintain figure readability, the 35 eligible subtypes with the highest maximum stage-specific mean primary developmental module scores were displayed. No additional minimum-cell threshold was imposed on individual stage-by-subtype combinations. White heatmap entries therefore indicate combinations for which no cells were available in the processed atlas. These analyses were descriptive, and individual cells were not treated as independent biological replicates.

### 2.11. Mouse Cortical Developmental Single-Cell Atlas Analysis

For descriptive cross-species comparison, wild-type cells from the GSE153162 mouse cortical developmental dataset were analyzed to characterize the cellular and developmental distribution of the BrainSpan-derived developmental signature and canonical clock-related genes during mouse corticogenesis. Only wild-type, non-perturbed samples were retained, whereas Fezf2 heterozygous and knockout samples were excluded. The retained dataset comprised 13 wild-type samples spanning E10, E11.5, E12.5, E13.5, E14.5, E15.5, E16, E17.5, E18, E18.5, P1, and P4. Developmental-stage annotations were curated from the original sample identifiers before stage-stratified analyses.

The initial wild-type dataset contained 82,415 cells and 27,998 genes. Quality control was performed separately within each sample. Cells were required to contain more than 300 detected genes, more than 500 total transcript counts, and less than 15% mitochondrial transcripts. To additionally remove sample-specific outliers, median-absolute-deviation filtering was applied independently within each sample using two-sided five-MAD limits for the numbers of detected genes and total counts and an upper five-MAD limit for mitochondrial transcript percentage. After quality-control filtering, 79,891 cells were retained.

Doublets were subsequently identified independently within each sample using Scrublet (version 0.2.3), with an expected doublet rate of 0.06. Predicted doublets were removed, yielding 77,322 cells for downstream analysis. Raw counts were normalized to 10,000 counts per cell and log1p transformed. Three thousand highly variable genes were selected using the Seurat v3 method, with sample identity specified as the batch variable. Highly variable genes were scaled and subjected to principal-component analysis using 50 components.

Sample-associated technical variation was adjusted in PCA space using Harmony with sample identity as the integration variable and a maximum of 20 Harmony iterations. A nearest-neighbor graph was constructed from the Harmony-adjusted representation using 20 neighbors and the first 40 principal components. UMAP was generated with min_dist = 0.4, and Leiden clustering was performed at a resolution of 0.8. Major cortical cell classes were assigned using available metadata together with canonical lineage-marker expression and included radial glia/neural progenitor cells, intermediate progenitors, excitatory neurons, interneurons, astrocytes, endothelial cells, microglia, pericytes, and oligodendrocyte-lineage cells. Cell-type composition was summarized descriptively as the proportion of retained cells assigned to each major cell class within each developmental stage.

For cross-species projection, the corrected BrainSpan-derived transferable signature comprised 1515 genes from the postnatal-rising turquoise primary developmental module with |kMEturquoise| ≥ 0.75. Human-to-mouse orthologs were obtained using g:Profiler g:Orth. To prevent duplicated contributions of human module-membership weights through paralogous mouse genes, only strict one-to-one mappings were retained. A mapping was accepted only when one human input gene mapped to exactly one mouse gene and that mouse target was associated with only one human input gene within the returned mapping set. Ambiguous one-to-many and many-to-one mappings were excluded rather than propagated with duplicated or partitioned weights. Of the 1515 human signature genes, 1157 had at least one mouse ortholog returned and 1128 satisfied the strict one-to-one criterion, corresponding to 74.46% unique-gene retention and 74.65% retention of the total absolute BrainSpan kMEturquoise weight. The original signed BrainSpan kMEturquoise coefficient was assigned once to each retained mouse ortholog and was not re-estimated in the mouse dataset. The complete retained mapping, excluded genes, and mapping audit are provided in Supplementary Table S4, whereas mapping-retention statistics and the scoring definition are summarized in Supplementary Table S5.

### 2.12. Prenatal Immune Activation Dataset Processing

Poly(I:C) was administered to pregnant dams at E12.5 in the original study, and dorsal telencephalon/developing cortex samples were subsequently collected at E12.5 + 6 h, E14.5, E17.5, and P0. Because treatment was administered at the dam level, the litter rather than individual offspring was treated as the biological experimental unit in the revised analysis. Sample-level metadata were linked to corresponding litter identifiers using the supplementary metadata from the original study. Raw RNA-seq counts from offspring originating from the same litter were summed to generate litter-level expression profiles before normalization, differential-expression analysis, and gene-signature scoring. Samples without a traceable litter identifier were excluded from litter-level inferential analyses. The numbers of litters and sequenced offspring, including offspring sex distribution, are summarized in Supplementary Table S9. Individual RNA integrity numbers were not available in the public metadata and were therefore not inferred. RNA-quality procedures reported in the original study are described as provided in the source dataset. Because all animals were exposed at E12.5, collection stage was intrinsically linked to elapsed time after exposure; accordingly, the analysis was interpreted as a post-exposure developmental trajectory rather than a comparison of independent gestational susceptibility windows.

The BrainSpan-derived developmental signature was transferred to GSE166376 using exactly the same strict one-to-one human-to-mouse ortholog mapping defined for the GSE153162 analysis. Thus, the cross-species reference consisted of 1128 uniquely mapped mouse orthologs derived from the corrected 1515-gene turquoise signature, with each retained mouse gene receiving the original BrainSpan kMEturquoise coefficient exactly once. No additional ortholog selection, paralog expansion, or weight re-estimation was performed using the MIA dataset. For each litter-level profile, the fixed-weight score was calculated using the same normalized weighted-expression definition described above.

### 2.13. Differential Expression and Interaction Analysis in Prenatal Immune Activation

Differential-expression analyses were performed on litter-level aggregated raw counts using a negative-binomial generalized linear modeling framework. Stage-specific MIA-versus-control contrasts were fitted separately at E12.5 + 6 h, E14.5, E17.5, and P0. To test whether treatment effects differed across subsequent collection stages, a likelihood-ratio test compared a full model containing collection stage, treatment, and their interaction with a reduced model containing collection stage and treatment only: full model, expression ~ stage + treatment + stage:treatment; reduced model, expression ~ stage + treatment. Benjamini–Hochberg correction was applied for multiple testing. The numbers of genes passing an FDR threshold were retained as descriptive summaries but were not used to rank treatment-response magnitude across stages because threshold-based DEG counts depend on biological replication, dispersion, and statistical power. Treatment magnitude across stages was instead characterized using distributions of shrunken MIA-versus-control log2 fold changes.

### 2.14. Litter-Level Clock-Related and BrainSpan-Derived Developmental Signature Scoring in Prenatal Immune Activation

Canonical core clock-related genes were evaluated as a secondary biological annotation. For each litter-level expression profile, detected core clock genes were standardized across litters and averaged to generate a core clock-gene expression score. The independently defined higher-confidence BrainSpan-derived developmental signature was transferred to GSE166376 through eligible mouse orthologs while retaining the original signed BrainSpan module-membership (kME) weights. Neither signature membership nor gene weights were re-estimated using the MIA dataset. Fixed-weight scores were calculated from litter-level variance-stabilized expression profiles. Linear models were used to evaluate the overall treatment effect after accounting for collection stage and to test the collection-stage-by-treatment interaction. Because GSE166376 comprises bulk developing cortical tissue, these scores were interpreted as aggregate tissue-level expression measures and cannot distinguish within-cell-state transcriptional regulation from developmental or treatment-associated changes in cellular composition.

### 2.15. Representative Gene Trajectory Analysis After Prenatal Immune Activation

Representative genes from the core clock, homeostatic/neurodevelopmental, and immune-response categories were selected a priori from predefined biological annotations rather than on the basis of differential-expression significance or visual inspection of treatment effects. Core clock-related genes were chosen to represent canonical clock components, whereas homeostatic/neurodevelopmental and immune-response genes provided biological context for neuronal maturation, metabolic regulation, and inflammatory signaling. Core clock genes included *Arntl*, *Clock*, *Npas2*, *Per1*, *Per2*, *Cry1*, *Nr1d1*, and *Rora*. Homeostatic and neurodevelopmental genes included *Nampt*, *Egr1*, *Eif4e*, *Mef2d*, and *Ube3a*. Immune-response genes included *Stat3*, *Socs3*, *Ifit1*, *Isg15*, and *Irf7*. Expression values were plotted across developmental stages, with individual samples shown as points and group-level trends displayed separately for control and MIA samples.

### 2.16. ASD Postmortem Brain Dataset Analysis

The GSE28521 postmortem brain dataset was used as an exploratory region-stratified boundary assessment to determine whether projected developmental and clock-related signatures showed detectable ASD–control differences in heterogeneous postmortem tissue. This analysis was not designed as an independent validation of the BrainSpan-derived developmental program or as a test of ASD-specific convergence. Expression data and metadata were retrieved and processed at the gene level. Samples were grouped by diagnosis and by the original GSE28521 brain-region annotation. Region labels were retained exactly as defined in the source dataset and included frontal cortex (F), temporal cortex (T), and cerebellum (C). Analyses were performed separately within each region to reduce confounding by regional transcriptional differences. Projected module scores were calculated for multiple gene sets, including the ASD synaptic excitation–inhibition gene set, BrainSpan-derived developmental signature, clock-output gene set, canonical core clock gene set, neuronal activity/homeostasis gene set, and top WGCNA hub-gene sets. Within each region, ASD-versus-control effect estimates were obtained using linear modeling. Effect estimates, 95% confidence intervals, and nominal *p* values were visualized using forest plots.

To evaluate the reproducibility of the BrainSpan-derived developmental module, we performed an independent computational replication using the human cortical developmental transcriptomic dataset GSE30272. Expression profiles were processed and annotated using the corresponding platform annotation file. BrainSpan-derived module genes were mapped to the external dataset, and module expression patterns were evaluated across developmental stages. This analysis was conducted independently of the discovery WGCNA.

### 2.17. Statistical Analysis

All statistical analyses were performed using R (version 4.5.0) and Python 3. Generalized additive models were used for continuous developmental modeling. Group comparisons were performed using Wilcoxon rank-sum tests or linear models, as appropriate. For co-expression network analysis, module–trait associations were assessed by correlation analysis with false-discovery-rate adjustment. Differential-expression analyses used negative-binomial generalized linear models and likelihood-ratio tests. Module-score analyses used linear models containing stage, treatment, and interaction terms. Multiple-testing correction was performed using the Benjamini–Hochberg method. Unless otherwise specified, FDR < 0.05 was considered statistically significant. Visualization was performed using standard R (version 4.5.0) and Python 3 plotting frameworks, including UMAP plots for single-cell visualization, dot plots for gene expression across cell classes, heatmaps for scaled expression and module scores, boxplots for group comparisons, PCA for global transcriptomic structure, and forest plots for region-stratified ASD effect estimates.

## 3. Results

### 3.1. Core Clock-Related Genes Undergo Structured Developmental Remodeling in the Human Frontal Cortex

**Figure 1:**
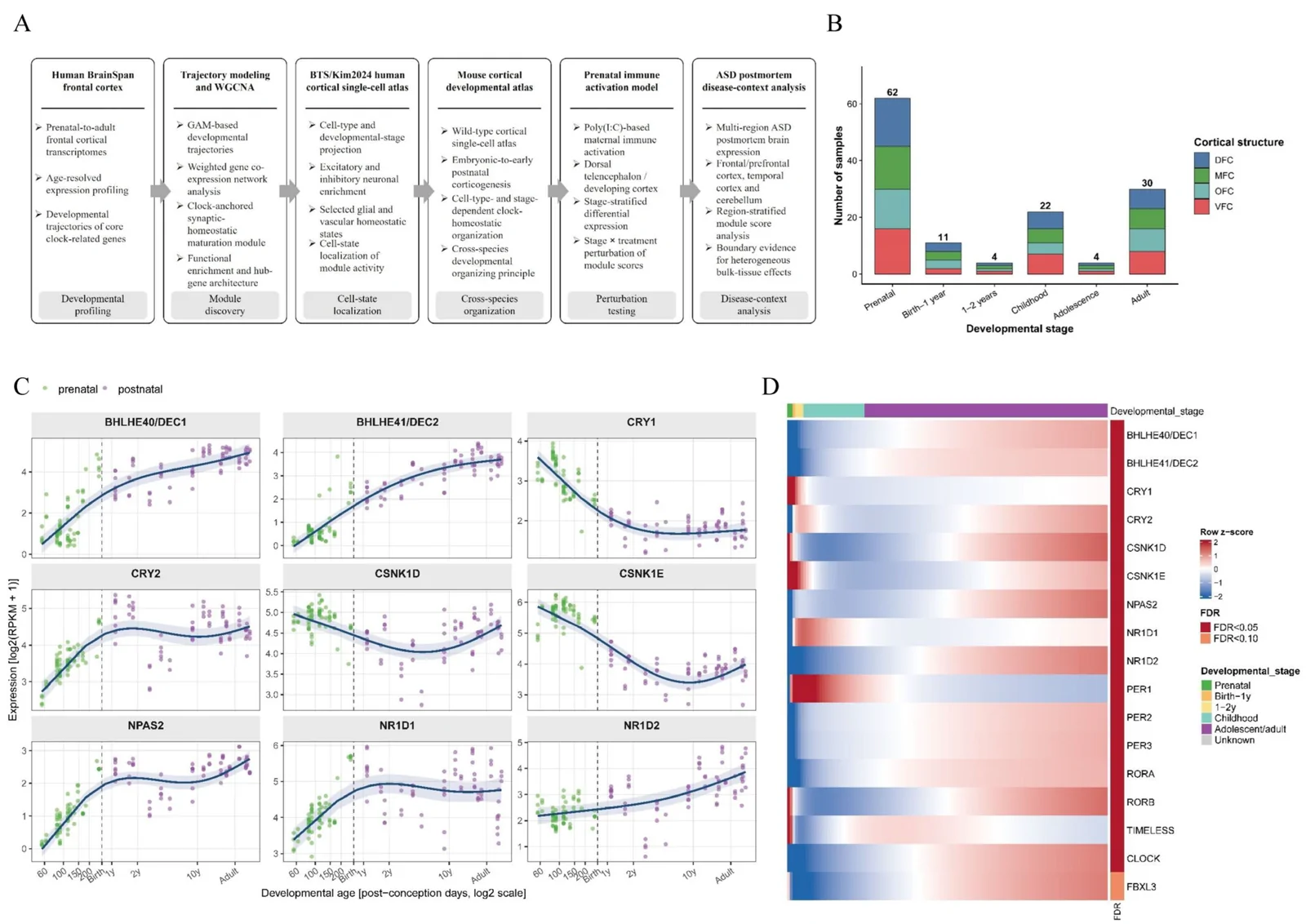
Developmental remodeling of core clock-related genes in the human frontal cortex. (**A**) Overview of the integrative study design. (**B**) Distribution of BrainSpan frontal cortical samples across developmental stages and cortical structures. Samples were grouped into prenatal, birth–1 year, 1–2 years, childhood, adolescence, and adult stages, with cortical structure annotations including dorsolateral frontal cortex, medial frontal cortex, orbital frontal cortex, and ventrolateral frontal cortex. (**C**) Representative developmental trajectories of core clock-related genes in the human frontal cortex. Gene-wise generalized additive models were fitted to log2(RPKM + 1) expression values using a thin-plate regression spline for log2-transformed post-conception age (k = 4), with sex and cortical structure included as fixed covariates and donor identity included as a random-effect smooth when repeated donor samples were available. Models were fitted using REML. Points indicate individual samples colored by prenatal or postnatal status. Solid curves indicate fitted trajectories, shaded regions indicate 95% confidence intervals, and the vertical dashed line marks birth. Representative genes illustrate postnatal-rising, prenatal-high, and biphasic/switch-like developmental patterns. (**D**) Age-ordered predicted expression heatmap of core clock-related genes. Predicted expression values were derived from generalized additive models and row-scaled for visualization. Columns represent age-ordered developmental prediction points, and rows represent clock-related genes. Side annotations indicate developmental stage and statistical significance based on FDR-adjusted age effects. The heatmap shows that clock-related genes segregate into distinct developmental trajectory classes rather than forming a uniform expression program.

To determine whether canonical clock-related genes are dynamically organized during human cortical development, we first established an integrative analytical framework centered on the BrainSpan human frontal cortex transcriptomic dataset. The study design combined age-resolved transcriptomic profiling, trajectory modeling, co-expression network discovery, cross-species single-cell projection, prenatal immune activation analysis, and neurodevelopmental disease-context assessment (Figure 1A). For the initial developmental analysis, frontal cortical samples were stratified across prenatal, early postnatal, childhood, adolescence, and adult periods and represented multiple frontal cortical structures, including dorsolateral, medial, orbital, and ventrolateral frontal cortex (Figure 1B).

We fitted gene-wise generalized additive models to BrainSpan frontal cortical samples, modeling expression as a smooth function of developmental age on a log2-transformed post-conception-day scale while adjusting for sex and cortical structure. Representative core clock and clock-regulatory genes exhibited highly significant age-dependent trajectories, but their direction and shape were markedly heterogeneous (Figure 1C). *BHLHE40* and *BHLHE41* showed progressive postnatal upregulation, consistent with greater engagement of clock-associated transcriptional repressors during later cortical maturation. *NPAS2* and *NR1D2* also displayed postnatal-rising patterns, suggesting that selected clock-related transcriptional regulators become increasingly incorporated into the maturing frontal cortical transcriptional program. By contrast, *CRY1* and *CSNK1E* showed prenatal-high trajectories, with higher fetal expression followed by postnatal attenuation. A third group, including *CRY2*, *CSNK1D*, and *NR1D1*, exhibited biphasic or switch-like patterns around the prenatal-to-postnatal transition. Thus, individual clock components are developmentally regulated in a gene-specific manner rather than moving as a uniform oscillator-like unit.

Age-ordered predicted expression profiles further confirmed that core clock-related genes segregated into distinct developmental trajectory classes (Figure 1D). Postnatal-rising genes included *BHLHE40*, *BHLHE41*, *NPAS2*, *NR1D2*, *RORA*, *RORB*, *CLOCK*, and *FBXL3*, whereas prenatal-enriched or early-high genes included *CRY1* and selected PER family members. *CRY2*, *CSNK1D*, *CSNK1E*, and *NR1D1* showed nonlinear transitions across birth and early postnatal development. These patterns demonstrate gene-specific developmental remodeling of clock-related transcript abundance rather than a uniform age-dependent shift. Because sampling time was not controlled, these trajectories do not inform within-day phase, amplitude, periodicity, or oscillator function. Overall, the human frontal cortex shows structured developmental remodeling of core clock-related gene expression, providing a rationale for network-level analyses of whether these genes are embedded within broader programs of synaptic maturation, metabolic regulation, and cellular maintenance.

### 3.2. WGCNA Identifies a Developmental Cortical Co-Expression Module Containing Clock-Related Genes and Enriched for Neuronal Maturation Processes

**Figure 2:**
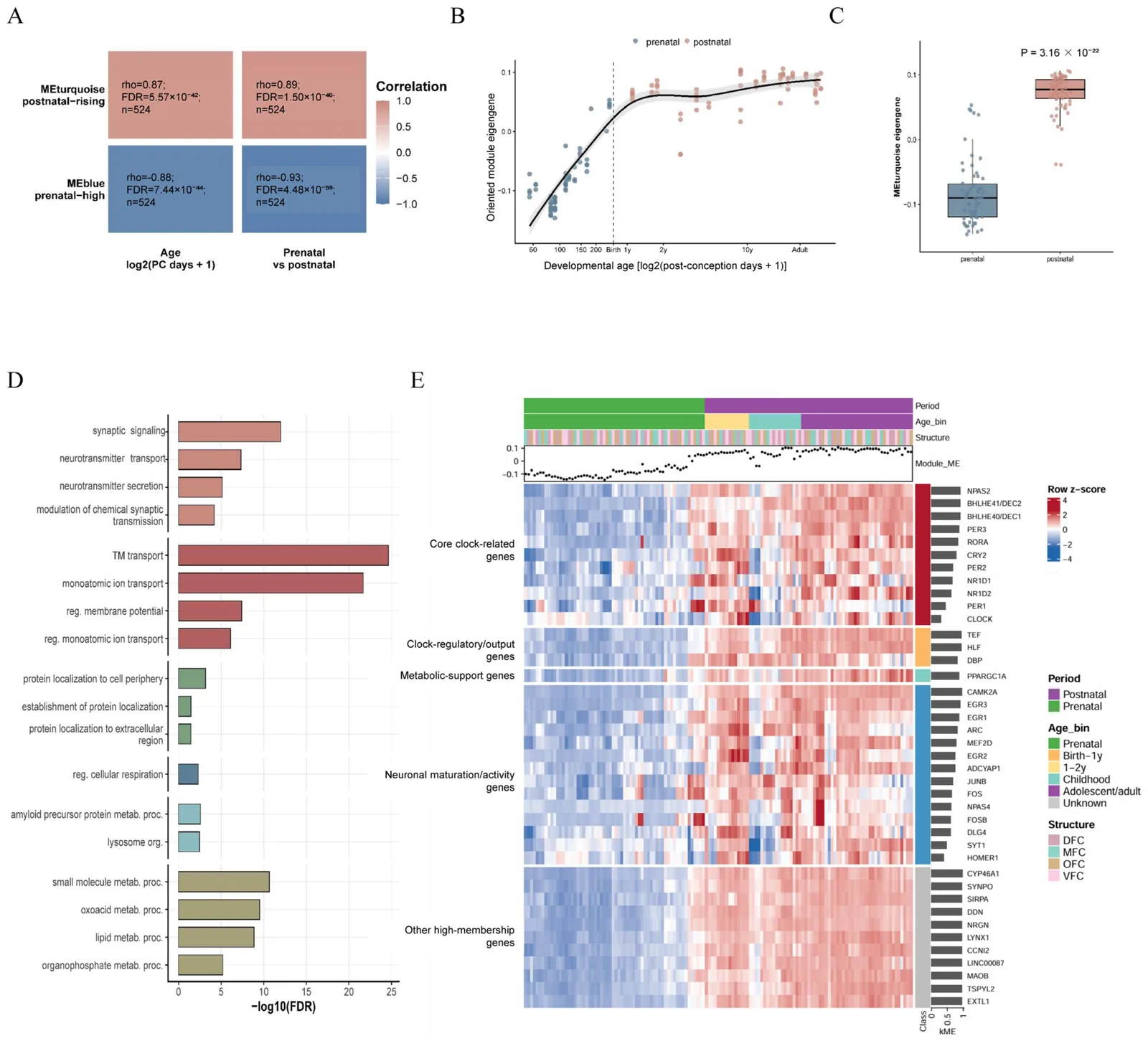
Identification of a BrainSpan-derived primary developmental module containing clock-related genes and enriched for neuronal maturation programs. (**A**) Module–trait correlation heatmap from weighted gene co-expression network analysis (WGCNA) of BrainSpan frontal cortical transcriptomes. The turquoise module showed a strong positive association with continuous developmental age (Spearman rho = 0.87, FDR-adjusted *p* = 5.57 × 10^−42^) and postnatal status (Spearman rho = 0.89, FDR-adjusted *p* = 1.50 × 10^−46^), whereas the blue module showed reciprocal prenatal-high developmental patterns (age: rho = −0.88, FDR-adjusted *p* = 7.44 × 10^−43^; prenatal/postnatal status: rho = −0.93, FDR-adjusted *p* = 4.48 × 10^−55^). All correlations were calculated using 524 BrainSpan frontal cortical samples. Values indicate Spearman correlation coefficients, with FDR-adjusted *p* values shown in each cell. (**B**) Developmental trajectory of the turquoise module eigengene across human frontal cortical development. Each point represents one of the 524 BrainSpan frontal cortical samples, colored according to prenatal or postnatal status. The *x*-axis shows developmental age on a log2-transformed post-conception day scale. The fitted curve indicates the smoothed developmental trajectory, and the vertical dashed line marks birth. (**C**) Comparison of turquoise module eigengene values between prenatal and postnatal samples. Boxplots show median and interquartile range with individual samples overlaid. Postnatal samples showed significantly higher module eigengene values than prenatal samples (Wilcoxon rank-sum test, *p* = 3.16 × 10^−22^). An adjusted linear model controlling for sex and cortical structure confirmed a significant postnatal increase (=0.156, *p* = 7.22 × 10^−46^). (**D**) Functional enrichment analysis of the selected module. Representative enriched biological processes are grouped into synaptic signaling, ion transport and membrane excitability, protein localization/proteostasis, cellular respiration, lysosomal organization, and metabolic processes. The *x*-axis indicates enrichment significance as −log10(FDR). The strongest enrichment signals involved synaptic signaling and ion transport-related processes, with additional enrichment for metabolic, respiratory, lysosomal, and proteostatic pathways. The robustness of these functional characteristics after removal of manually curated clock-related genes was further evaluated by sensitivity analysis (Supplementary Figure S1). (**E**) Hub-gene architecture of the selected module. The heatmap shows row-scaled expression of representative high-membership genes across BrainSpan frontal cortical samples ordered by developmental age. Top annotations indicate developmental period, age bin, cortical structure, and module eigengene values. Genes are grouped into canonical clock-related genes, clock-output transcription factors, metabolic-support genes, neuronal maturation/activity-associated genes, and other high-membership genes. Canonical clock-gene membership is shown as a biological annotation and is not interpreted as equivalent to hub status.

Having established gene-specific developmental remodeling of core clock-related genes in the human frontal cortex, we next asked whether these genes are embedded within broader developmental co-expression architectures. We therefore performed weighted gene co-expression network analysis (WGCNA) using BrainSpan frontal cortical transcriptomes. Among the detected modules, two showed the strongest reciprocal associations with developmental age and the prenatal-to-postnatal transition. The turquoise module was positively correlated with continuous developmental age and postnatal status, whereas the blue module showed the opposite prenatal-high pattern (Figure 2A). Because WGCNA module colors are arbitrary, interpretation was not based on color assignment. Instead, the primary developmental module was selected exclusively from its developmental associations, including its correlation with continuous age and its prenatal-to-postnatal transition pattern. Biological annotation was performed only after module selection to characterize clock-associated, neuronal, metabolic, and regulatory features. The selected module was therefore interpreted as a BrainSpan-derived primary developmental module rather than a predefined clock-gene program.

The eigengene of the selected developmental module showed a pronounced postnatal-rising trajectory across human frontal cortical development (Figure 2B). Eigengene values were low prenatally, increased sharply around the prenatal-to-postnatal transition, and remained elevated across later postnatal periods. The eigengene was strongly positively associated with continuous developmental age (Spearman rho = 0.87, FDR-adjusted *p* = 5.57 × 10^−42^, n = 524), and the age-dependent trajectory remained significant in a generalized additive model adjusted for sex and cortical structure (GAM age effect *p* < 2 × 10^−16^). Consistently, postnatal samples had markedly higher eigengene values than prenatal samples (Wilcoxon rank-sum test, *p* = 3.16 × 10^−22^; adjusted linear model, β = 0.156, *p* = 7.22 × 10^−46^; postnatal–prenatal mean difference = 0.15; Figure 2C). These results indicate that the module captures a coordinated transcriptional program associated with postnatal frontal cortical maturation. Because BrainSpan bulk cortical profiles span a broad developmental interval, we next tested whether the module was driven primarily by shared developmental and sample-level effects. After residualization for developmental age, donor identity, sex, and cortical structure, module-membership estimates within the predefined turquoise gene set showed moderate concordance with the original WGCNA-derived values (Spearman ρ = 0.418; Supplementary Figure S4A). Overlap among the top-ranked genes was limited, with 18%, 25%, and 29% overlap for the top 50, 100, and 200 genes, respectively (Supplementary Figure S4B). These findings indicate partial internal concordance of gene-membership estimates after covariate adjustment, together with substantial reorganization of hub ranking. Because the analysis retained the original module membership rather than reconstructing a genome-wide residual network, it does not establish preservation of the original co-expression architecture independently of developmental or compositional effects.

Functional enrichment analysis of the BrainSpan-derived developmental module revealed biological programs related to synaptic signaling, ion transport, membrane excitability, and metabolic regulation (Figure 2D). Additional enriched categories included protein localization, lysosomal organization, cellular respiration, small-molecule metabolism, oxoacid metabolism, lipid metabolism, and organophosphate metabolism, implicating proteostatic, bioenergetic, and metabolic processes that support mature neuronal activity. *Clock-associated* biological processes were also represented, consistent with the integration of circadian-related regulators into broader developmental transcriptional networks. Thus, the module encompasses neuronal maturation, metabolic support, regulatory processes, and clock-associated transcriptional regulation within a broader developmental cortical program. The turquoise module contained 12 of the 18 predefined canonical clock-related genes represented in the WGCNA universe. Although this corresponded to an odds ratio of 2.27, enrichment did not reach statistical significance after multiple-testing correction (one-sided Fisher’s exact *p* = 0.073; BH-FDR = 0.220; Supplementary Table S6). Accordingly, the turquoise module is interpreted as a developmental cortical module containing multiple canonical clock-related genes rather than as a statistically clock-enriched module.

To further define the primary developmental module as a reproducible analytical entity, we retained all genes assigned to the selected WGCNA module as the full primary module (n = 2818 genes), preserving the original gene membership and signed module-membership coefficients (kME) (Supplementary Table S1). Module connectivity showed a broad distribution of membership strengths within the original BrainSpan developmental network (Supplementary Figure S3A). Higher-confidence representations of the same module were then generated using |kME| thresholds of 0.75 and 0.85, corresponding to a transferable signature and a highly connected hub subset, respectively (Supplementary Tables S2 and S3). These hierarchical representations did not redefine the developmental module; rather, they captured progressively higher levels of network connectivity within the same program. Functional enrichment analyses showed that major biological features were preserved across the full module, the |kME| ≥ 0.75 signature, and the |kME| ≥ 0.85 hub subset, including axon guidance, neuron projection development, forebrain development, RNA processing, and translational regulation (Supplementary Figure S3B). These findings support the robustness of the developmental program across different levels of network connectivity.

We next examined canonical clock-related and other biologically annotated genes across the primary developmental module architecture. Clock-related genes, including NPAS2, BHLHE40, BHLHE41, PER family members, RORA, CRY2, NR1D1/2, CLOCK, and FBXL3, showed varying degrees of module membership. Canonical clock-gene identity was therefore not interpreted as equivalent to hub status, and genes with only moderate module membership were not designated as major network hubs (Figure 2E). The same module contained genes associated with neuronal activity and synaptic maturation, including *CAMK2A*, *EGR1*–3, *ARC*, *JUNB*, *FOS*/*FOSB*, *NPAS4*, *DLG4*, *SYT1*, and *HOMER1*. Because several of these genes are activity-responsive immediate-early genes, their presence is interpreted as an annotation of neuronal state rather than evidence for clock-specific regulatory organization.

### 3.3. Single-Cell Projection Localizes the BrainSpan-Derived Primary Developmental Module to Postnatal Neuronal Maturation States

To determine the cellular context of the BrainSpan-derived primary developmental module, we projected the fixed postnatal-rising turquoise-module signature onto the human cortical developmental single-cell atlas. The transferable signature comprised 1515 genes with |kMEturquoise| ≥ 0.75, of which 1191 were detected as unique features in the BTS atlas, corresponding to 78.6% gene retention and 78.8% retention of the absolute BrainSpan kME weights. The original BTS cell annotations and UMAP coordinates were retained, and the BrainSpan-derived weights were not re-estimated in the single-cell dataset.

**Figure 3:**
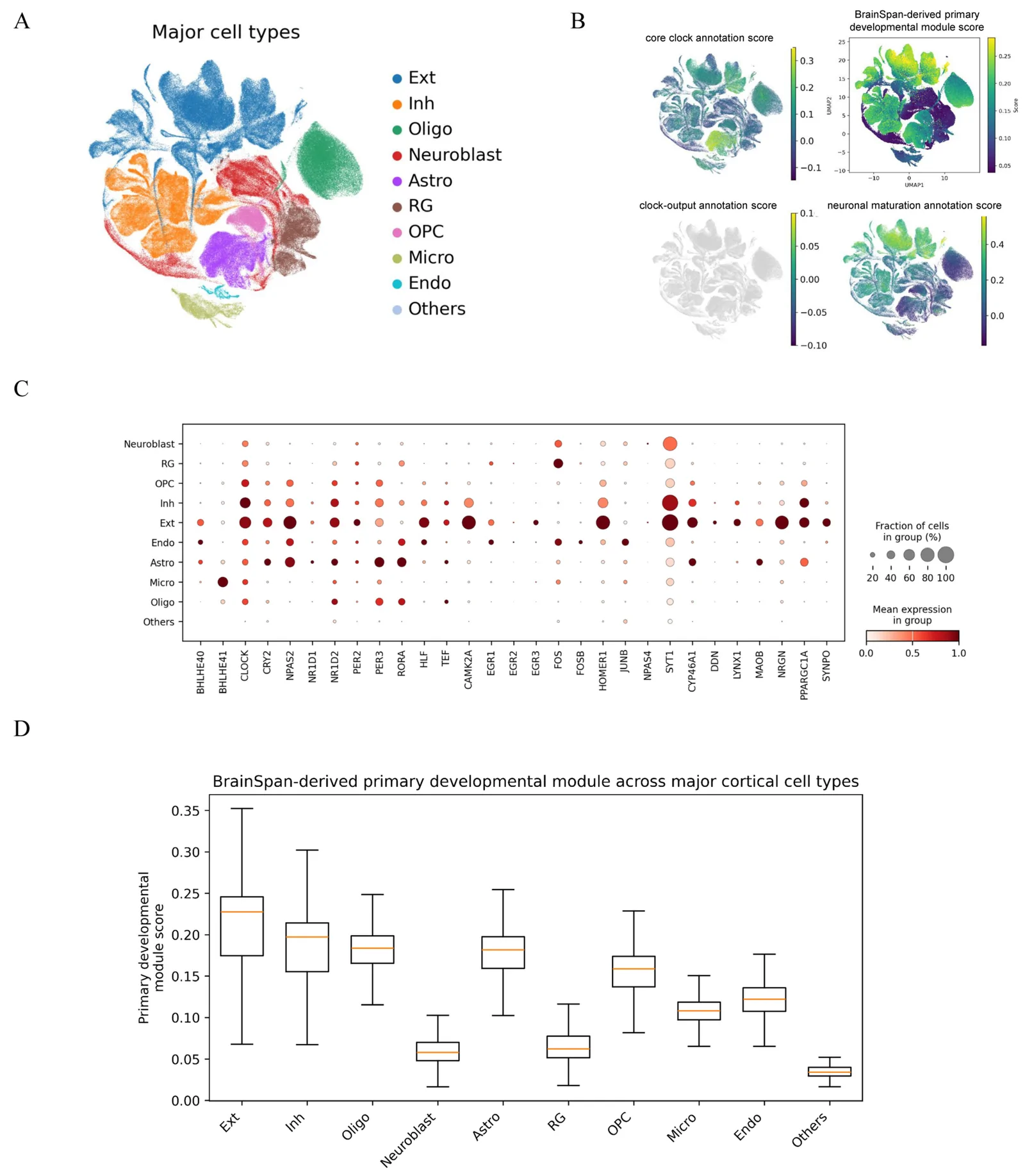
Cellular localization of the corrected BrainSpan-derived primary developmental module in the human cortical developmental atlas. (**A**) UMAP representation of the human cortical developmental single-cell atlas using the original BTS atlas coordinates and major cell-type annotations. Major populations include excitatory neurons (Ext), inhibitory neurons (Inh), oligodendrocytes (Oligo), neuroblasts, astrocytes (Astro), radial glia (RG), oligodendrocyte precursor cells (OPC), microglia (Micro), endothelial cells (Endo), and other minor populations. (**B**) UMAP projections of independent gene-program scores. The BrainSpan-derived primary developmental module was defined as the postnatal-rising turquoise module and transferred using the fixed |kMEturquoise| ≥ 0.75 signature. Of the 1515 transferable genes, 1191 were detected in the BTS atlas. Canonical core-clock, clock-output, and neuronal-maturation scores were calculated independently as secondary biological annotations. The corrected primary-module score showed a structured and non-uniform cellular distribution distinct from the broader distribution of clock-related gene expression. (**C**) Dot plot showing representative clock-related and neuronal maturation-associated genes across major cortical cell types. Dot size represents the fraction of cells expressing each gene, whereas color indicates scaled mean expression within the corresponding population. These genes are displayed as biological annotations and were not used to redefine the primary developmental module in the single-cell dataset. (**D**) Distribution of the fixed-weight BrainSpan-derived primary developmental module score across major cortical cell types. Boxes indicate the median and interquartile range, with whiskers representing the corresponding cell-level score distributions. Scores were highest in excitatory and inhibitory neurons and were also relatively elevated in oligodendrocytes, astrocytes, and OPCs, whereas substantially lower scores were observed in neuroblasts and radial glia. These distributions localize expression of the fixed BrainSpan-derived signature across cortical cell states but do not establish preservation of the original bulk co-expression architecture within individual cell populations.

Projection of the corrected signature revealed a strongly non-uniform distribution across major cortical cell classes (Figure 3B,D). The highest scores were observed in excitatory neurons (median = 0.227) and inhibitory neurons (median = 0.197), with relatively high scores also present in oligodendrocytes (median = 0.184), astrocytes (median = 0.182), and oligodendrocyte precursor cells (median = 0.159). By contrast, radial glia and neuroblasts showed substantially lower scores (medians = 0.062 and 0.058, respectively). Thus, in contrast to the reciprocal prenatal-high program, the corrected postnatal-rising BrainSpan module was preferentially represented in differentiated neuronal and selected glial states rather than in early progenitor populations. Canonical clock-related genes remained more broadly distributed across cortical populations, indicating that expression of the developmental module is not simply equivalent to the cellular distribution of individual clock genes (Figure 3B,C).

**Figure 4:**
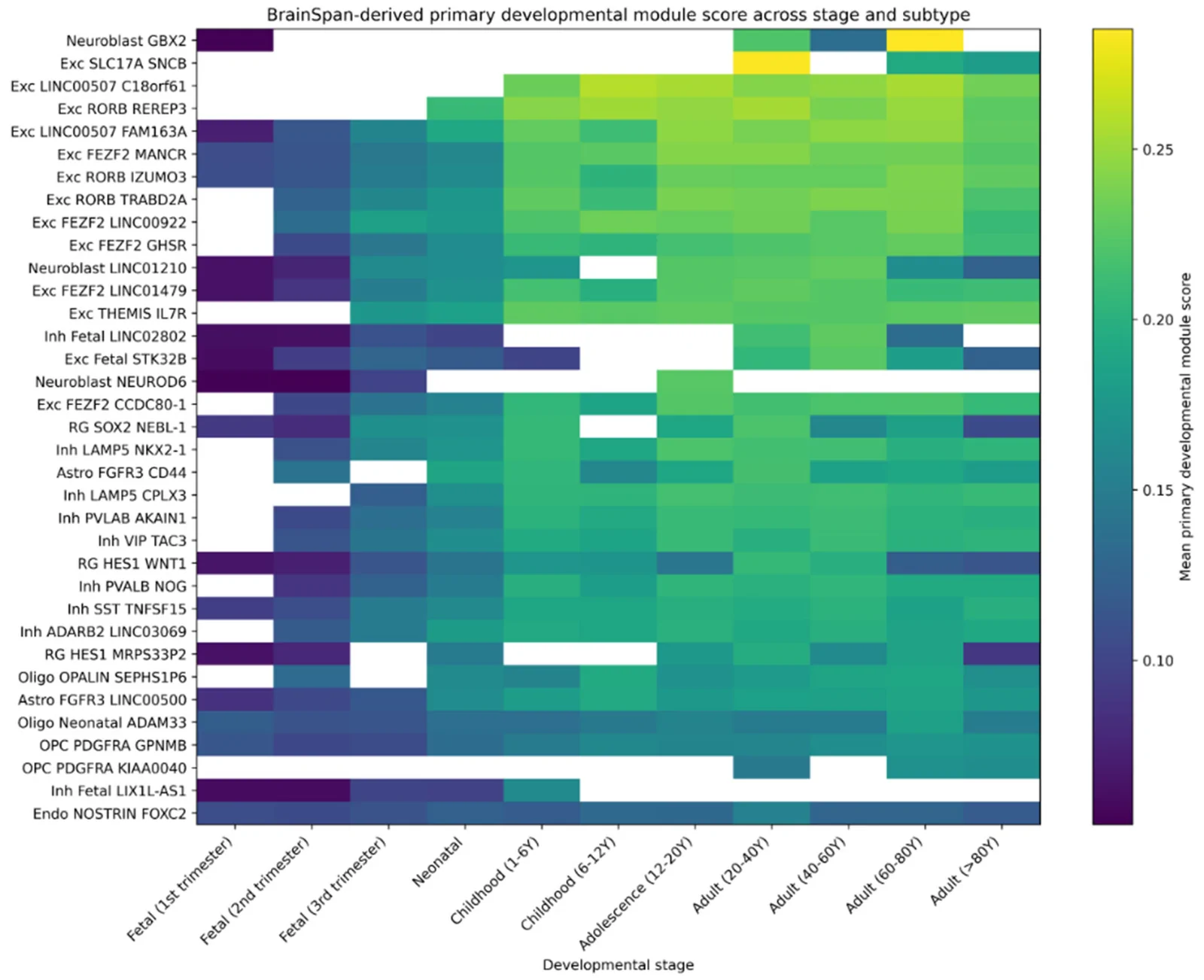
Developmental and cell-state distribution of the corrected BrainSpan-derived primary developmental signature in the human cortical single-cell atlas. Heatmap showing the mean fixed-weight primary developmental module score across annotated developmental-stage-by-cell-subtype combinations. Rows represent cortical cell subtypes and columns represent developmental stages from fetal development through adulthood. Subtypes represented by at least 300 cells across the full analyzed atlas were eligible for inclusion, and the 35 eligible subtypes with the highest maximum stage-specific mean scores are displayed. Color intensity indicates the mean primary developmental module score within each available stage–subtype combination. White cells indicate combinations for which no cells were available in the processed atlas; no additional per-combination minimum-cell threshold was applied. The analysis is descriptive, and individual cells were not treated as independent biological replicates.

The stage-by-subtype analysis further supported this developmental organization (Figure 4). Across many excitatory and inhibitory neuronal subtypes, primary-module scores were relatively low during the first and second fetal trimesters, increased during late fetal and neonatal development, and remained comparatively elevated across childhood, adolescence, and adulthood. Several astrocytic and oligodendrocyte-lineage states showed a similar postnatal increase, although the magnitude and timing varied across subtypes. These patterns are therefore consistent with the postnatal-rising trajectory of the bulk BrainSpan turquoise module while also showing substantial cell-state heterogeneity. Because these projections summarize expression of a fixed bulk-derived gene signature, they are interpreted descriptively and do not establish preservation of the original BrainSpan co-expression topology within individual cell populations.

### 3.4. A Mouse Developmental Single-Cell Atlas Provides a Cross-Species View of Clock-Related and Developmental Signature Expression

**Figure 5:**
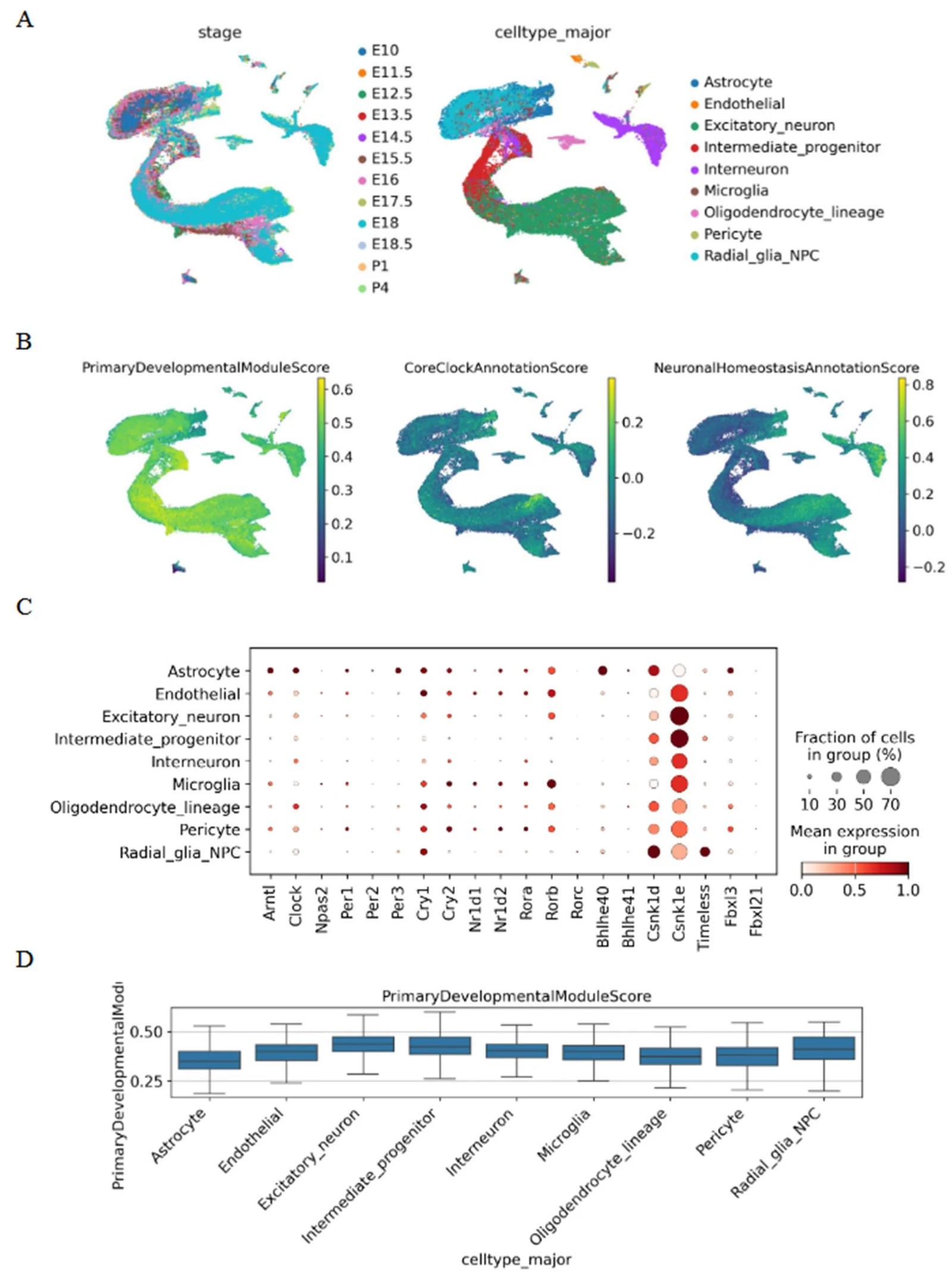
Cross-species transfer of the BrainSpan-derived primary developmental module to the developing mouse cortex. (**A**) UMAP representation of wild-type GSE153162 cortical single-cell transcriptomes colored by developmental stage (**left**) and major cell type (**right**). Developmental stages span E10, E11.5, E12.5, E13.5, E14.5, E15.5, E16, E17.5, E18, E18.5, P1, and P4. Major cell classes include astrocytes, endothelial cells, excitatory neurons, intermediate progenitors, interneurons, microglia, oligodendrocyte-lineage cells, pericytes, and radial glia/neural progenitor cells (Radial_glia_NPC). Developmental-stage metadata were re-curated from the original sample identifiers, eliminating the previously unresolved stage category. (**B**) UMAP visualization of the BrainSpan-derived primary developmental module score and two independently defined secondary annotation scores. The primary developmental module was transferred from the human BrainSpan WGCNA to the mouse dataset through human-to-mouse ortholog mapping while retaining the original BrainSpan-derived kME weights. The core clock gene-set score and neuronal maturation gene-set score were calculated separately and are shown only as secondary biological annotations. Their partially distinct spatial distributions demonstrate that these gene sets are not interchangeable representations of the primary developmental program. (**C**) Dot plot showing the expression of representative canonical clock and clock-regulatory genes across major mouse cortical cell types. Dot size represents the fraction of cells expressing each gene within the corresponding cell class, whereas color intensity represents mean normalized expression. These genes are displayed to provide biological context for clock-related expression and were not used independently to define or validate preservation of the transferred primary developmental module. (**D**) Distribution of the fixed-weight BrainSpan-derived primary developmental module score across major mouse cortical cell types. Boxes indicate the median and interquartile range, with whiskers representing the corresponding score distribution. The transferred BrainSpan-derived signature was broadly expressed across cortical lineages, with relatively higher scores in excitatory neurons, intermediate progenitors, and radial glia/neural progenitor populations.

To provide a cross-species developmental context, we projected the fixed BrainSpan-derived signature and independently defined clock-related annotations onto wild-type cells from the GSE153162 mouse cortical atlas. This analysis compared their stage- and cell-type distributions during mouse corticogenesis rather than testing preservation of the human BrainSpan network topology. After quality control, integration, clustering, and marker-based annotation, UMAP visualization resolved a structured developmental landscape spanning embryonic and early postnatal stages from E10 to P4 (Figure 5A). The BrainSpan-derived primary developmental module was transferred to the mouse dataset through human-to-mouse ortholog mapping while preserving the original BrainSpan-derived gene weights. Projection analysis demonstrated that the transferred developmental module showed broad representation across the developing mouse cortex, with a spatial distribution distinct from independently defined core clock and neuronal maturation annotation scores (Figure 5B). These annotations were consistent with canonical lineage markers and provided an independent cross-species framework for evaluating clock-related transcriptional organization during cortical development.

These annotations were supported by canonical lineage marker expression patterns across major cortical populations (Figure 5C). Quantification across major cortical populations revealed variable module scores among different cell classes, with relatively higher scores observed in excitatory neurons, intermediate progenitors, and radial glia/neural progenitor populations (Figure 5D). These findings indicate that the BrainSpan-derived module represents a conserved developmental transcriptional program that is distributed across multiple cortical lineages rather than being restricted to a single mature neuronal population.

**Figure 6:**
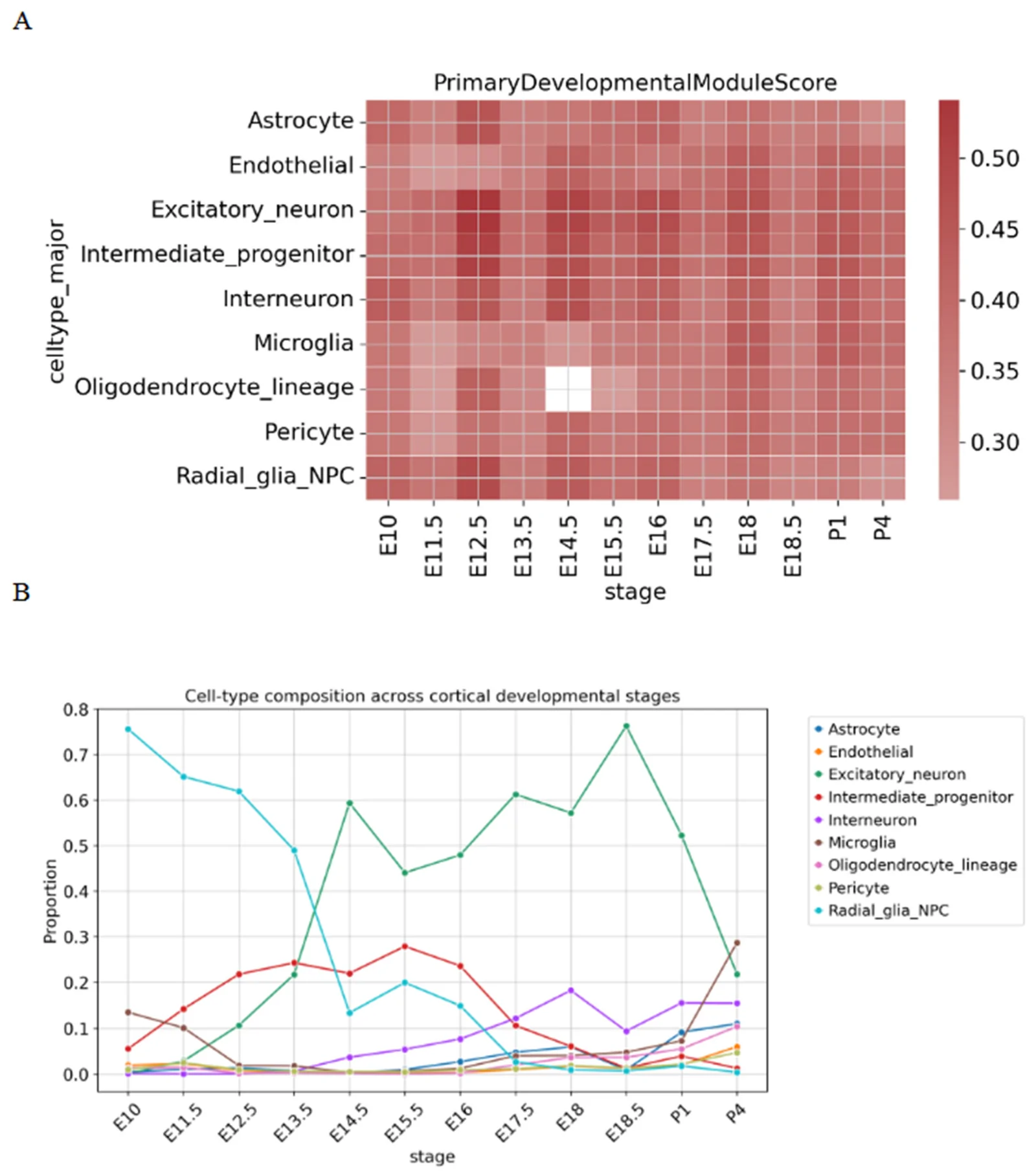
Developmental and cellular organization of the transferred BrainSpan-derived primary developmental module in mouse cortex. (**A**) Heatmap showing the mean BrainSpan-derived primary developmental module score across developmental-stage-by-major-cell-type combinations in the mouse cortical single-cell atlas. Rows indicate major cortical cell classes and columns indicate developmental stages from E10 to P4. Color intensity represents the mean fixed-weight primary developmental module score within each stage–cell-type combination. White cells indicate stage–cell-type combinations with insufficient or unavailable observations. The heterogeneous pattern across developmental stages and cellular lineages indicates that the transferred human developmental program is deployed in a stage- and cell-state-dependent manner rather than following a uniform temporal trajectory across the entire cortex. (**B**) Cell-type composition across mouse cortical developmental stages. For each developmental stage, the proportion of cells assigned to each major cortical cell class is shown. This analysis provides a descriptive assessment of developmental changes in cellular composition and is presented separately from the primary developmental module score to distinguish changes in cell-type abundance from within-state expression of the transferred developmental program.

We next examined the organization of canonical clock genes by cell identity and developmental stage in the mouse cortical atlas. Dot-plot analysis showed that core clock-related genes were broadly but non-uniformly expressed across cortical populations (Figure 6A). Several clock-associated regulators, including *Arntl*, *Clock*, *Cry1*, *Csnk1d*, and *Csnk1e*, were detectable across radial glia/neural progenitors, intermediate progenitors, excitatory neurons, interneurons, microglia, vascular-associated cells, and glial-lineage populations. Other genes, including *Npas2*, *Per2*, *Nr1d1*, and *Rora*, showed more heterogeneous or restricted expression. Stage-wise analysis further demonstrated that both mean expression and the fraction of expressing cells varied across embryonic and early postnatal stages (Figure 6B), indicating that clock-related gene expression changes with developmental stage rather than remaining constant throughout corticogenesis.

To assess the reproducibility of the developmental pattern identified in the BrainSpan cohort, we performed an independent computational replication using a separate human cortical developmental transcriptomic dataset (GSE30272). Projection of BrainSpan-derived primary developmental module genes onto this independent dataset reproduced an age-associated expression trajectory during cortical maturation (Supplementary Figure S2). This result supports the reproducibility of the developmental transcriptional signature across independent human cortical datasets rather than a cohort-specific observation.

Together, these analyses show that canonical clock-related genes are embedded within the developmental transcriptional architecture of the mouse cortex, with substantial heterogeneity across developmental stages and cellular states. The mouse single-cell atlas provides comparative evidence for broad representation of clock-related genes across cortical lineages and dynamic regulation during maturation. This cross-species correspondence, however, should not be interpreted as equivalence in circadian phase, oscillation amplitude, environmental entrainment, or temporal regulation between mice and humans, given fundamental differences in circadian biology and ecological adaptation between nocturnal rodents and diurnal humans.

### 3.5. Prenatal Immune Activation Produces Stage-Resolved Litter-Level Transcriptomic Responses Following E12.5 Exposure

**Figure 7:**
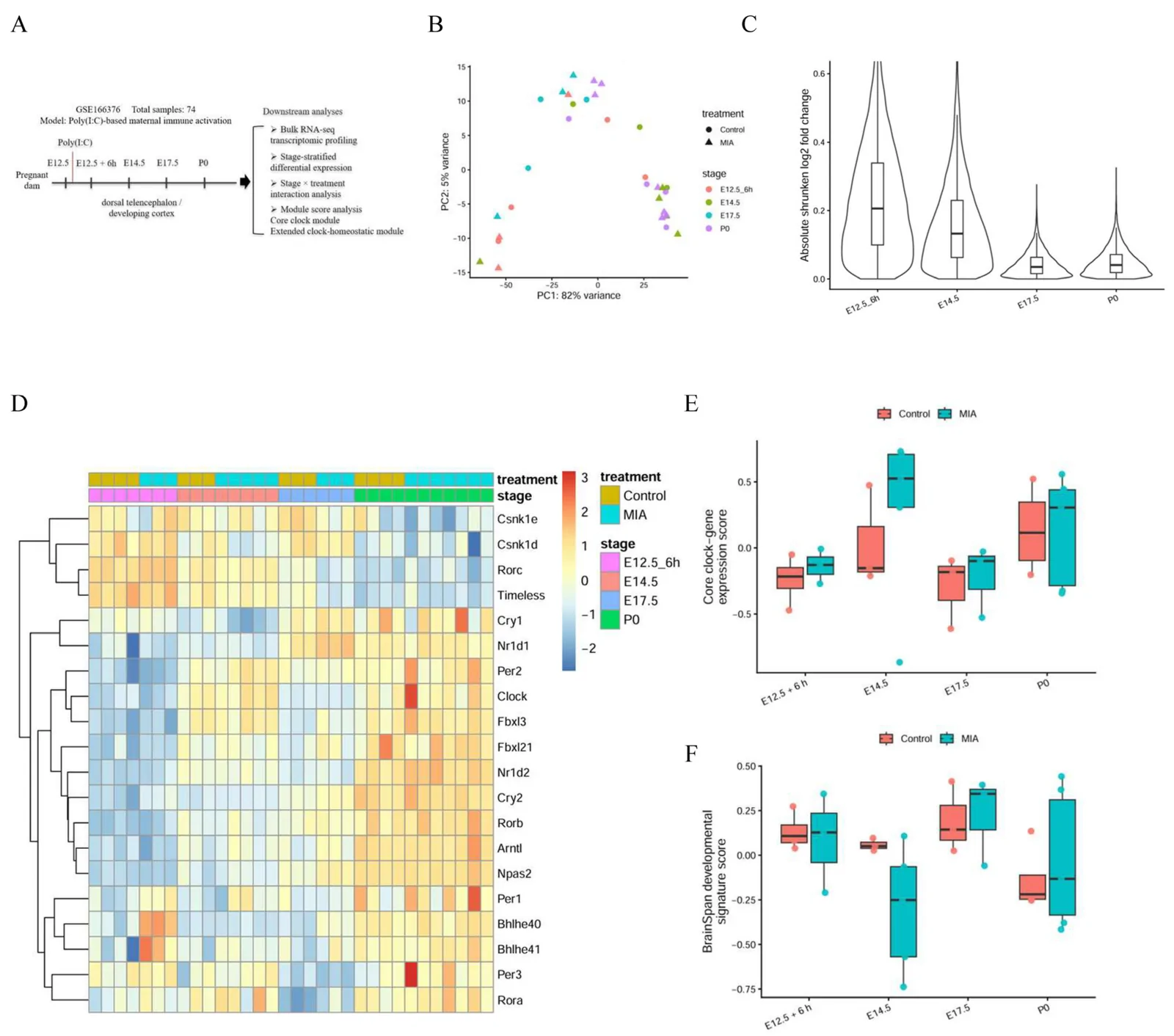
Litter-aware stage-resolved transcriptional responses following E12.5 prenatal immune activation. (**A**) Experimental design and revised analytical framework for GSE166376. Poly(I:C) or saline was administered to pregnant dams at E12.5, and offspring dorsal telencephalon/developing cortex was collected at E12.5 + 6 h, E14.5, E17.5, and P0. Offspring RNA-seq profiles were linked to litter of origin, and raw counts were aggregated within litter before inferential analysis. (**B**) Principal component analysis of litter-level variance-stabilized transcriptomic profiles. Each point represents an independent litter-level profile, colored by collection stage and shaped by treatment group. (**C**) Genome-wide distributions of litter-level MIA-versus-control treatment effect sizes across collection stages. Absolute shrunken log2 fold changes are shown. Threshold-based DEG counts were not used to rank response magnitude across stages. (**D**) Litter-level expression heatmap of canonical core clock-related genes. Columns represent independent litter-level profiles and rows represent genes; expression values are shown as row z-scores. (**E**) Litter-level core clock-gene expression score across collection stages and treatment groups. The stage effect showed a trend (F = 2.62, *p* = 0.071), whereas neither the overall MIA effect (β = 0.092, *p* = 0.506) nor the stage-by-treatment interaction (F = 0.159, *p* = 0.923) was significant. (**F**) Litter-level fixed-weight BrainSpan-derived developmental signature score. The corrected 1515-gene turquoise signature was transferred using the same strict one-to-one human-to-mouse ortholog mapping used for GSE153162. A total of 1128 unique ortholog pairs were retained, and each mouse ortholog received the original BrainSpan kMEturquoise weight exactly once.

To characterize developmental transcriptional responses to prenatal inflammatory challenge, we reanalyzed the GSE166376 maternal immune activation dataset using litter as the biological experimental unit. The original dataset comprised 74 offspring dorsal telencephalon/developing cortex RNA-seq samples collected at E12.5 + 6 h, E14.5, E17.5, and P0 after maternal saline or Poly(I:C) exposure at E12.5. Because treatment was administered to pregnant dams, individual offspring were not considered statistically independent biological replicates in the revised analysis. Offspring samples were therefore linked to their litter of origin, and raw RNA-seq counts from offspring within the same litter were aggregated before normalization and inferential analysis (Figure 7A).

After litter-level aggregation and gene filtering, principal component analysis of variance-stabilized expression profiles showed that collection stage remained the dominant source of global transcriptomic variation (Figure 7B). Litter-level profiles separated primarily by developmental stage, with additional within-stage variation between control and MIA profiles. Thus, transcriptional responses after E12.5 Poly(I:C) exposure occur against a strong background of ongoing cortical developmental change and should be interpreted in relation to both collection stage and elapsed time after exposure.

Stage-stratified litter-level contrasts further showed that genome-wide treatment-effect distributions differed across subsequent collection points (Figure 7C). Because the number of genes passing a fixed FDR threshold depends on the number of independent biological replicates, dispersion, and statistical power, DEG counts were treated descriptively and were not used to rank response magnitude across stages. Instead, we compared distributions of shrunken MIA-versus-control log2 fold changes. Median absolute effect sizes were larger at E12.5 + 6 h and E14.5 (median |log2FC| = 0.206 and 0.133, respectively) and more attenuated at E17.5 and P0 (0.035 and 0.041, respectively). The litter-aware analysis therefore supports a temporally heterogeneous cortical transcriptional response after E12.5 Poly(I:C) exposure but does not support designation of E17.5 as a uniquely maximal or preferentially vulnerable collection stage.

We next examined canonical clock-related gene expression separately from the independently defined BrainSpan-derived developmental signature. All 20 canonical core clock-related genes were represented in the processed expression profiles. A litter-level heatmap showed pronounced developmental organization of clock-related transcript abundance across the four collection stages, with descriptive control–MIA differences for selected genes superimposed on this developmental structure (Figure 7D). At the aggregate gene-set level, the core clock-gene expression score showed a trend toward variation across collection stages (F = 2.62, *p* = 0.071; Figure 7E). After accounting for collection stage, however, MIA exposure was not associated with a significant overall change in the core clock-gene expression score (β = 0.092, *p* = 0.506), and the collection-stage-by-treatment interaction was also not significant (F = 0.159, *p* = 0.923). Thus, canonical clock-related transcripts retain marked developmental organization in the developing cortex, but the litter-aware analysis provides no evidence for a global or collection-stage-specific MIA-associated shift in the aggregate core clock-gene expression score.

To directly evaluate the independently defined developmental program under the corrected experimental-unit framework, we transferred the predefined higher-confidence BrainSpan-derived developmental signature to litter-level GSE166376 profiles using eligible mouse orthologs while retaining the original BrainSpan-derived signed module-membership weights. Gene membership and weights were not re-estimated in the MIA dataset. After accounting for collection stage, no significant overall MIA-associated change in the transferred developmental signature was detected (β = −0.053, 95% CI −0.258 to 0.151, *p* = 0.597; Figure 7F), and the collection-stage-by-treatment interaction was likewise not significant (F = 1.17, *p* = 0.343). Stage-specific treatment contrasts were also non-significant at E12.5 + 6 h (β = −0.045, *p* = 0.774), E14.5 (β = −0.361, *p* = 0.135), E17.5 (β = 0.032, *p* = 0.871), and P0 (β = 0.117, *p* = 0.573). Thus, after accounting for litter-level dependence, GSE166376 provides no robust statistical evidence that E12.5 Poly(I:C) exposure alters aggregate expression of the transferred BrainSpan-derived developmental signature.

**Figure 8:**
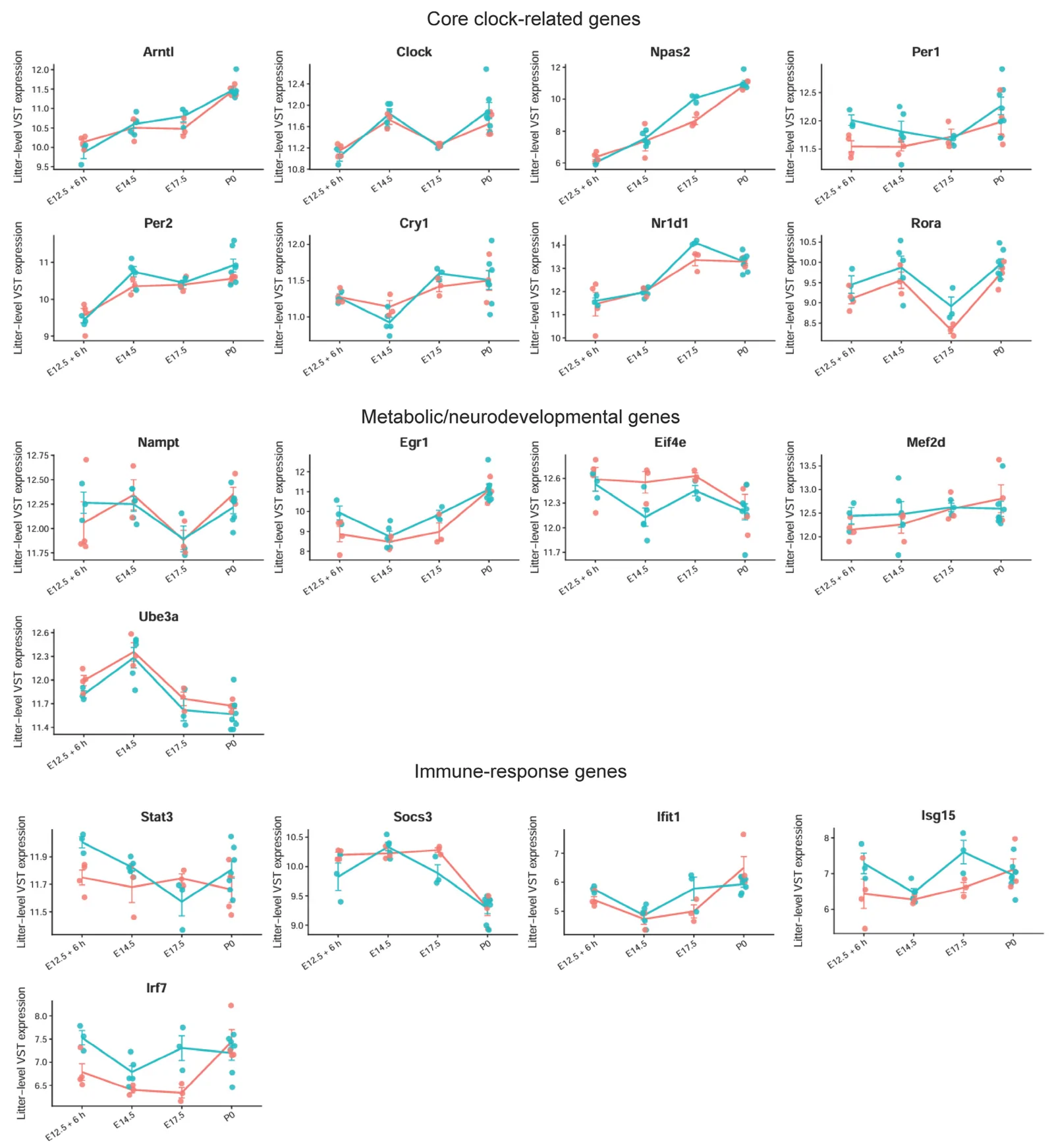
Representative litter-level expression trajectories of clock-related and secondary response genes following prenatal immune activation. Expression trajectories of representative canonical clock-related, metabolic/neurodevelopmental, and immune-response genes are shown across E12.5 + 6 h, E14.5, E17.5, and P0 following E12.5 Poly(I:C) exposure. Raw offspring counts originating from the same litter were aggregated before normalization, and individual points therefore represent independent litter-level expression profiles. Lines indicate group mean trajectories for control and MIA litters. Canonical clock-related genes include *Arntl*, *Clock*, *Npas2*, *Per1*, *Per2*, *Cry1*, *Nr1d1*, and *Rora*; metabolic/neurodevelopmental genes include *Nampt*, *Egr1*, *Eif4e*, *Mef2d*, and *Ube3a*; and immune-response genes include *Stat3*, *Socs3*, *Ifit1*, *Isg15*, and *Irf7*. Representative genes were selected a priori from predefined biological categories and were not chosen according to the magnitude or direction of Poly(I:C)-associated changes. These trajectories are presented as descriptive biological context rather than evidence for selective gene regulation, cell-type-specific vulnerability, or perturbation of the BrainSpan-derived developmental network.

Representative litter-level gene trajectories provided secondary biological context for the temporal transcriptional response after E12.5 Poly(I:C) exposure (Figure 8). Canonical clock-related genes, including *Arntl*, *Clock*, *Npas2*, *Per1*, *Per2*, *Cry1*, *Nr1d1*, and *Rora*, retained pronounced developmental trajectories across E12.5 + 6 h, E14.5, E17.5, and P0, with descriptive control–MIA differences for selected genes. Metabolic and neurodevelopmental genes, including *Nampt*, *Egr1*, *Eif4e*, *Mef2d*, and *Ube3a*, and immune-response genes, including *Stat3*, *Socs3*, *Ifit1*, *Isg15*, and *Irf7*, similarly showed temporally structured litter-level expression patterns. These trajectories illustrate biological components of the bulk cortical response and were not interpreted as evidence for perturbation of the BrainSpan-derived co-expression network or regulation within specific neuronal, glial, or clock-related cell states. Because GSE166376 comprises bulk developing cortical tissue, observed differences may reflect transcriptional-state changes, developmental shifts in cellular composition, or both.

**Figure 9:**
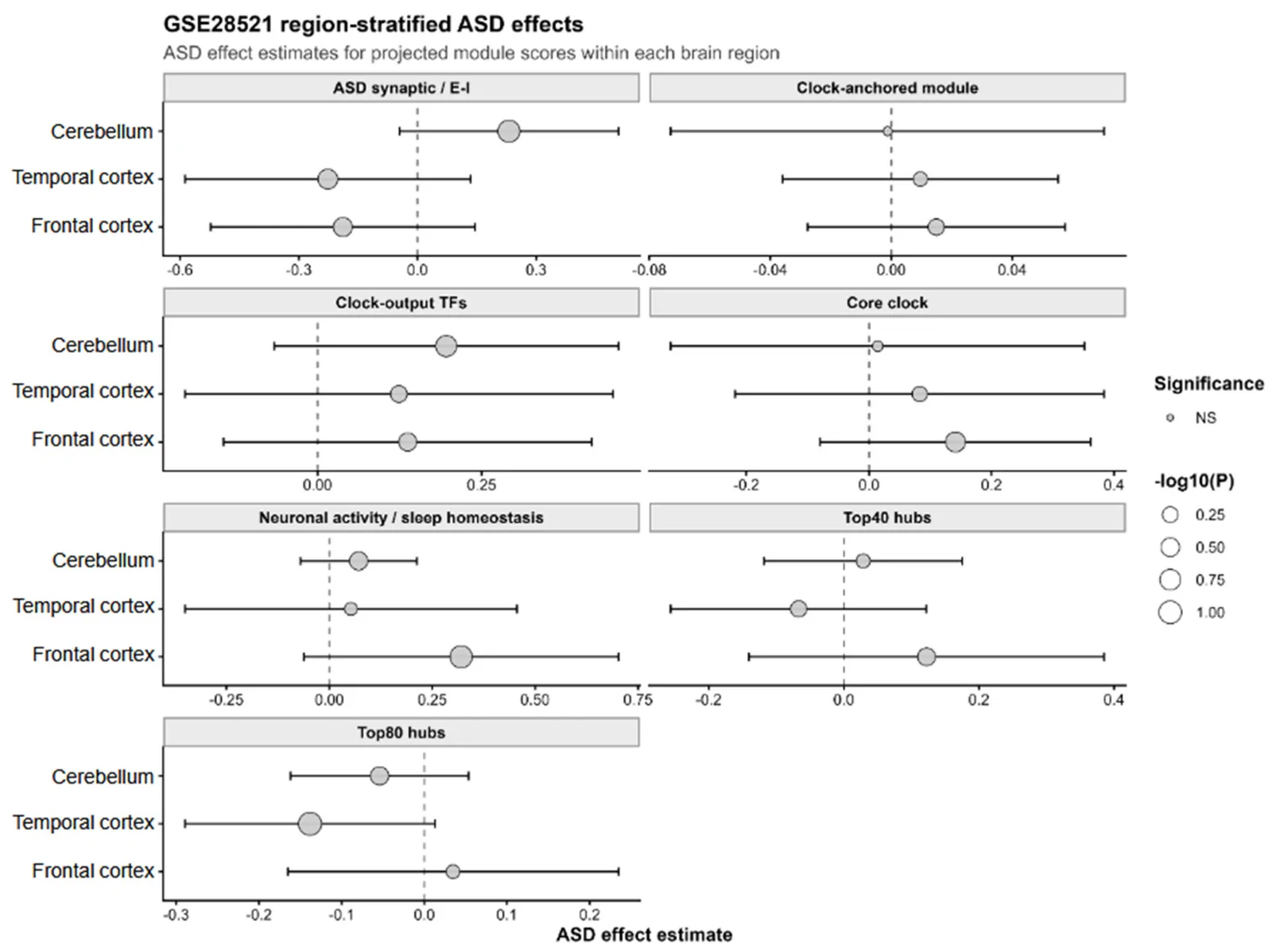
Exploratory region-stratified ASD–control estimates for projected developmental and clock-related signatures in GSE28521. Forest plots show ASD-versus-control effect estimates and 95% confidence intervals separately within the original GSE28521 anatomical categories: frontal cortex, temporal cortex, and cerebellum. The BrainSpan-derived developmental signature showed near-zero estimates with confidence intervals crossing zero across all examined regions. Related core clock, clock-output, neuronal activity/homeostasis, synaptic/E-I, and hub-gene sets likewise showed weak or region-variable estimates without a consistent cross-region pattern. GSE28521 was therefore treated as an exploratory negative-result boundary assessment and was not used as evidence for ASD-specific convergence, developmental restriction, cell-type confinement, or signal dilution.

As an exploratory disease-context boundary assessment, we examined developmental and clock-related signatures in the multi-region GSE28521 ASD postmortem cohort (Figure 9). ASD-versus-control effect estimates were calculated separately within frontal cortex, temporal cortex, and cerebellum while retaining the original GSE28521 anatomical labels. The BrainSpan-derived developmental signature yielded effect estimates close to zero across all three regions, with 95% confidence intervals crossing zero in each case. Core clock- and clock-output-related signatures likewise showed weak and imprecise estimates, and the remaining annotation sets showed no consistent cross-region pattern. GSE28521 therefore provided no statistical evidence for a reproducible ASD-associated alteration of the BrainSpan-derived developmental signature or related clock-associated gene sets. These null findings were treated as an exploratory boundary assessment and were not used to infer developmental restriction, cell-type confinement, signal dilution, or ASD-specific convergence.

Collectively, the litter-aware GSE166376 analysis indicates that the transcriptional consequences of a single E12.5 Poly(I:C) exposure evolve across subsequent cortical development. Genome-wide treatment-effect distributions differed across collection stages, with larger absolute effects at the earlier E12.5 + 6 h and E14.5 collection points and more attenuated distributions at E17.5 and P0. In contrast, neither the aggregate core clock-gene expression score nor the independently transferred BrainSpan-derived developmental signature showed a significant overall MIA effect or collection-stage-by-treatment interaction after litter was treated as the biological experimental unit. The MIA dataset therefore provides a stage-resolved perturbational context for developmental and clock-related cortical transcription but does not establish selective disruption of the BrainSpan-derived developmental program, altered within-module connectivity, or cell-type-specific regulation. Moreover, because collection stage is intrinsically linked to elapsed time after the single E12.5 exposure, these findings describe a post-exposure developmental trajectory rather than distinct gestational windows of susceptibility.

## 4. Discussion

In this study, we identified a BrainSpan-derived developmental co-expression framework in which clock-related genes are embedded within broader cortical maturation-associated transcriptional changes. Core clock-related genes in the frontal cortex did not behave as a single coordinated block but instead followed postnatal-rising, prenatal-high, and nonlinear developmental trajectories. WGCNA identified a module enriched for synaptic signaling, neurotransmitter handling, ion transport, membrane excitability, metabolic support, and proteostatic functions. Residual sensitivity analysis showed moderate concordance between original and covariate-adjusted module-membership estimates within the predefined turquoise gene set (Spearman ρ = 0.418), whereas highly ranked genes were substantially reorganized. This sensitivity analysis therefore supports only partial internal concordance under covariate adjustment and should not be interpreted as evidence that the original BrainSpan co-expression network is preserved independently of developmental age or cellular composition. We accordingly interpret the BrainSpan-derived module as a developmental co-expression framework associated with cortical maturation rather than a fixed, cell-state-independent regulatory network. Single-cell analyses further localized this framework to defined cortical cell states, whereas canonical clock-related expression was more broadly distributed across cell classes.

A key interpretive constraint is that the present datasets do not establish circadian rhythmicity per se. BrainSpan is a foundational developmental transcriptome resource, but it was not generated using time-of-day-controlled serial sampling. Likewise, the human cortical single-cell atlases used for projection were designed to resolve cell states, lineage progression, and developmental timing across prenatal and postnatal life rather than repeated zeitgeber phases within the same developmental epoch [29,30,39,40]. The same limitation applies to the mouse cortical atlas and the Poly(I:C)-based MIA dataset. Accordingly, developmental remodeling of *ARNTL*, *CLOCK*, *NPAS2*, PER-family, CRY-family, NR1D-family, and ROR-family transcripts should be interpreted as developmental deployment of clock-associated regulators rather than direct evidence of rhythmic transcription.

These findings align with a broader view of clock genes as homeostatic transcriptional regulators whose neural functions extend beyond daily timekeeping. Molecular clock components are present in neuronal and glial compartments and have been linked to neuronal activity, astrocytic and microglial function, cortical excitability, and synaptic plasticity [11,41,42,43,44]. *Clock-related* regulators also intersect with metabolic regulation, mitochondrial oxidative metabolism, NAD+ cycling, redox homeostasis, stress adaptation, and immune signaling [12,13,14,15,45].

The biological identity of the BrainSpan module is therefore best described as a developmental cortical co-expression framework containing clock-related genes rather than a fixed regulatory or canonical circadian module. Importantly, sensitivity analysis excluding manually curated clock-related genes preserved the dominant functional features of the module, indicating that the developmental co-expression program reflects broader transcriptional organization rather than an artifact of initial gene-set construction.

A canonical circadian oscillator module would be expected to center on clock-loop architecture, whereas the strongest functional enrichments observed here involved synaptic signaling, neurotransmitter transport and secretion, transmembrane ion transport, regulation of membrane potential, cellular respiration, lysosomal biology, and small-molecule or lipid metabolism. This combination is consistent with a transcriptional program that supports the emergence and stabilization of mature postnatal cortical activity states, in which increased synaptic throughput requires coordinated control of excitability, ATP production, oxidative burden, protein turnover, and membrane trafficking. Clock-related genes are therefore best viewed as one annotated component of a broader developmental cortical co-expression framework whose dominant biological features involve neuronal maturation, synaptic signaling, metabolic support, and cellular maintenance.

An important implication of the postnatal-rising trajectory is that this module should not be interpreted as the initiating lesion in neurodevelopmental disorders whose pathogenic processes may begin during fetal development. Genetic and environmental risks for disorders such as ASD and schizophrenia can act during early corticogenesis, when cortical cell specification, neuronal differentiation, and circuit assembly are being established [1,26,27,28,31]. Our findings are more consistent with a temporally layered model in which early developmental perturbations alter the trajectory or regulatory competence of emerging cortical cell states, whereas the clock-related maturation program becomes increasingly engaged later as neuronal signaling, membrane excitability, metabolic demand, and proteostatic requirements increase. In this framework, clock-related genes may act as secondary modifiers or downstream maturation effectors that influence how earlier developmental disturbances are expressed, consolidated, or compensated for during postnatal circuit maturation rather than serving as universal primary drivers of disease initiation. This interpretation is also compatible with the prenatal immune-activation analysis, in which the perturbation precedes later developmental changes in clock-related and maturation-associated transcriptional programs. Because the present analyses are cross-sectional and correlative, however, they cannot determine whether individual clock-related regulators mediate, amplify, compensate for, or simply mark downstream consequences of earlier developmental perturbation. Longitudinal and perturbational studies will be required to distinguish among these possibilities.

The single-cell analyses provide complementary information on the cellular distribution of the BrainSpan-derived signature but do not validate the bulk WGCNA topology within individual cell types. Importantly, the revised analysis separates the distribution of canonical clock-related genes from localization of the BrainSpan-derived primary developmental module. Clock-related genes were detectable across multiple cortical populations, whereas the developmental module showed a more restricted cell-state pattern, arguing against the interpretation that the WGCNA signal simply reflects generalized clock-gene expression. Human cortical development is accompanied by major shifts in neuronal, progenitor, glial, and vascular states across the prenatal-to-postnatal transition [29]. A bulk postnatal-rising module could therefore reflect increased neuronal representation, maturation of neuronal transcriptional states, or both. Single-cell projection helps refine this ambiguity: the core clock gene-set score was broadly distributed across cortical cell classes, whereas the BrainSpan-derived primary developmental module was more enriched in postnatal excitatory and inhibitory neuronal states, with additional signals in selected astrocytic, endothelial, and other homeostatic compartments. This separation indicates that the projected signature is not uniformly distributed across cell classes while leaving open whether the original gene–gene covariance structure is preserved within specific states. The most balanced interpretation is that bulk WGCNA captured a cell-state-weighted developmental program shaped by both transcriptional state and cellular composition, and that single-cell projection strengthens the neuronal-homeostatic interpretation.

The mouse developmental atlas provides comparative evidence that clock-related genes are embedded within cortical developmental programs, but several qualifications are essential. Mice are predominantly nocturnal, whereas humans are predominantly diurnal; accordingly, coupling of the circadian system to behavioral state, feeding, body temperature, light exposure, and other environmental synchronizing signals differs substantially between the two species. Although the core transcriptional–translational clock architecture is evolutionarily conserved in mammals, conservation of molecular components does not imply conservation of circadian phase, amplitude, or tissue-specific entrainment [46,47]. Circadian transcripts in extra-SCN brain regions can show substantial phase differences between mouse and human tissues, reflecting differences in temporal niche and coupling of local clocks to systemic signals. The present mouse data should therefore be interpreted as supporting cross-species correspondence in the developmental deployment and cell-type distribution of clock-related genes rather than equivalence of their daily rhythmic organization [46].

Developmental timing introduces a second layer of divergence. Human cortical maturation is markedly prolonged relative to mouse development, and apparently corresponding prenatal or postnatal stages are not exact biological equivalents. Evolutionary divergence in cis-regulatory architecture, transcription-factor occupancy, cell-state composition, and coupling between clock-related regulators and neuronal activity may also alter when and where orthologous genes participate in cortical maturation. Such divergence could contribute to species-specific developmental phenotypes and limit direct extrapolation from mouse developmental states to human neurodevelopmental disorders. Cross-species projection was deliberately restricted to strict one-to-one orthologs to avoid duplicated paralog weighting. Although this conservative mapping retained approximately three quarters of the human transferable signature and its total absolute BrainSpan module-membership weight, the resulting scores should be interpreted as expression-level projections rather than evidence that the human module topology is conserved in mouse cortical states. Accordingly, the mouse analysis supports cross-species correspondence in the developmental and cell-state distribution of the transferred signature, but does not establish conservation of gene–gene covariance, hub hierarchy, identical developmental-stage alignment, or circadian timing. Determining which components of this organization are conserved versus evolutionarily specialized will require phase-controlled, stage-matched, and cell-type-resolved comparative studies across species.

The MIA analysis provides a stage-resolved perturbational context after a single prenatal immune challenge, but the litter-aware reanalysis requires conservative interpretation. In GSE166376, Poly(I:C) was administered only at E12.5, whereas transcriptomes were collected at E12.5 + 6 h, E14.5, E17.5, and P0. Collection stage is therefore intrinsically linked to elapsed time after exposure, and these data cannot identify independent gestational windows of susceptibility. After the litter, rather than individual offspring, was treated as the biological experimental unit, developmental stage remained the dominant organizer of transcriptomic variation, and genome-wide treatment-effect distributions differed across subsequent collection points. Larger absolute effects were observed at the earlier E12.5 + 6 h and E14.5 collection points, whereas effect-size distributions were more attenuated at E17.5 and P0. Importantly, neither the aggregate core clock-gene expression score nor the independently transferred BrainSpan-derived developmental signature showed a significant overall MIA effect or collection-stage-by-treatment interaction. The results therefore support a temporally evolving cortical transcriptional response after E12.5 maternal immune activation but do not demonstrate selective disruption of the BrainSpan-derived developmental program or a preferentially vulnerable developmental stage. Representative clock-related, metabolic, neurodevelopmental, and immune-response genes nevertheless showed temporally structured bulk-expression patterns after prenatal immune challenge. Variation in *Stat3*, *Socs3*, *Ifit1*, *Isg15*, and *Irf7* is consistent with inflammatory and interferon-related responses expected after Poly(I:C) exposure, whereas changes in genes related to neuronal activity and metabolic regulation provide broader biological context for the cortical response. These observations remain compatible with previous evidence that prenatal immune activation can produce timing-, brain-region-, and cell-type-dependent effects on fetal brain development [31,33,48]. Because GSE166376 comprises bulk developing cortical tissue, however, the present data cannot distinguish transcriptional regulation within specific neuronal, progenitor, or glial populations from developmental or treatment-associated changes in cellular composition. Potential links among inflammatory signaling, metabolic stress, activity-dependent transcription, and clock-related regulation should therefore be considered mechanistic hypotheses rather than evidence that these processes converge on or disrupt the BrainSpan-derived co-expression network. Litter-aware, cell-type-resolved MIA studies will be required to determine whether particular cortical cell states show selective transcriptional vulnerability after prenatal immune challenge.

ASD is etiologically and molecularly heterogeneous, and large-scale genomic and transcriptomic studies have shown that disease risk converges on multiple developmental and neuronal functional programs rather than a single molecular pathway [1]. Cell-type-resolved studies further indicate that ASD-associated molecular alterations can differ substantially across neuronal and glial populations [5,6]. In this context, the GSE28521 analysis should be interpreted strictly as an exploratory boundary assessment rather than evidence supporting ASD relevance of the BrainSpan-derived developmental program. Region-stratified estimates for the BrainSpan-derived developmental signature were close to zero, with confidence intervals crossing zero in frontal cortex, temporal cortex, and cerebellum; related clock-associated signatures were similarly weak and imprecise. Thus, the present data provide no evidence for a reproducible ASD-associated alteration of the developmental signature. These null findings cannot be attributed to signal dilution in bulk tissue, restriction of the program to earlier developmental periods, or confinement to specific cell states because none of these explanations was directly tested in GSE28521. The ASD postmortem analysis therefore defines an important boundary of the current study rather than serving as a validation dataset. Future studies using developmental-stage-resolved human ASD cohorts, cell-type-resolved transcriptomic datasets, or genetically defined ASD models will be needed to determine whether the BrainSpan-derived developmental program converges with ASD biology in specific developmental or cellular contexts.

This study has several limitations. All analyzed data are cross-sectional and derived from public resources. None of the datasets were time-of-day controlled; rhythmic transcription therefore cannot be inferred. WGCNA is correlative and cannot establish causal regulatory hierarchy. Accordingly, the temporal ordering observed here does not establish that the postnatal-rising module lies upstream of disease initiation, nor can it distinguish a causal modifier from a compensatory or downstream transcriptional response. Bulk modules inevitably conflate transcriptional state with cell composition, and single-cell projection localizes module scores without proving that the bulk-defined network operates identically within each cell type. Cross-species matching is approximate; the nocturnal–diurnal distinction between mice and humans, differences in developmental tempo, and species-specific circadian phase and entrainment limit direct inference of conserved temporal regulation from orthologous gene-expression patterns. The ASD postmortem dataset is further limited by sample size, tissue heterogeneity, and platform age. Finally, the study remains inferential in the absence of direct electrophysiological, metabolic, or perturbational validation. Transcriptomic measurements also reflect steady-state RNA abundance and do not directly report protein abundance, post-translational regulation, subcellular localization, or functional activity of clock-related factors. Developmental changes in transcript levels therefore indicate transcriptional remodeling, whereas protein-level dynamics may be further shaped by protein stability, degradation, phosphorylation, and cellular compartmentalization. The present findings should accordingly be interpreted as evidence for developmental organization of clock-related transcriptional programs rather than direct demonstration of corresponding functional protein changes.

Timed developmental sampling across defined zeitgeber phases will be required to determine whether the identified module is exclusively developmentally organized or also becomes rhythmic as cortical maturation proceeds. Human cortical organoids or assembloids could be used to perturb *ARNTL*, *CLOCK*, *NPAS2*, *NR1D1*, *NR1D2*, and *RORA*, alone or in combination with Poly(I:C)-related inflammatory signaling, to test whether the BrainSpan-derived primary developmental module lies causally downstream of clock regulators or is simply co-expressed with them. Single-cell MIA profiling could identify whether interferon-responsive microglia, radial glia, excitatory neurons, or inhibitory lineages preferentially carry the perturbational response. Parallel analyses in genetically defined ASD models, including monogenic syndromic models with established cortical developmental phenotypes, may provide a more mechanistically aligned framework for future validation of developmental-program vulnerability. Transcriptomic findings should ultimately be linked to function through synaptic electrophysiology, calcium imaging, mitochondrial respiration, redox assays, proteostasis/lysosome measurements, and, where possible, ASD cohort stratification by sleep/circadian phenotype.

In summary, these findings define a BrainSpan-derived cortical maturation framework in which clock-related genes are organized by developmental stage and cell state. Prenatal Poly(I:C) exposure provided an external stage-resolved perturbational context but did not demonstrate selective disruption of the transferred developmental signature, whereas exploratory ASD postmortem analysis provided no evidence of ASD-specific convergence.

## Data Availability

All datasets analyzed in this study are publicly available from established data repositories or brain atlas resources. Human developmental frontal cortical transcriptomic data and matched sample metadata were obtained from the BrainSpan Atlas of the Developing Human Brain. The human cortical developmental single-cell atlas used for cell-type and developmental-stage projection was obtained from the Brain Transcriptome Single-cell (BTS) Atlas reported by Kim et al. (2024) [39]. Processed BTS atlas files, including the AnnData object, Seurat object, CellTypist model, and neurological disorder risk-gene plots, were downloaded from Zenodo record 14177002, version v1.0.1; DOI: 10.5281/zenodo.14177002. Wild-type mouse cortical developmental single-cell transcriptomic data were obtained from GEO under accession GSE153162. Maternal immune activation transcriptomic data were obtained from GEO under accession GSE166376. Autism spectrum disorder postmortem brain expression data were obtained from GEO under accession GSE28521.

## Supplementary Materials

The following supporting information can be downloaded at: https://www.mdpi.com/article/10.3390/genes17091107/s1, Supplementary Figure S1. Sensitivity analysis evaluating the robustness of the BrainSpan-derived primary developmental module independent of predefined gene-set curation. (A) Contribution of manually curated clock-related genes to the selected WGCNA module. Overlap between module genes and predefined curated clock-related gene sets was quantified. The majority of module genes were independent of the original curated annotations. (B) Functional enrichment analysis of the complete BrainSpan-derived primary developmental module. Representative enriched biological processes include synaptic signaling, neurotransmitter transport, ion transport, metabolic regulation and neuronal homeostatic functions. (C) Functional enrichment analysis after removing genes overlapping with manually curated clock-related gene sets. The remaining module genes retained similar enrichment patterns, indicating that the biological identity of the module was not dependent on predefined gene-set selection. Supplementary Figure S2. Independent computational replication of the developmental trajectory of the BrainSpan-derived developmental program. (A) Developmental age distribution of the independent human cortical transcriptomic cohort GSE30272. (B) Distribution of gene-level developmental associations for BrainSpan-derived primary developmental module genes in the GSE30272 cohort. (C) Developmental trajectory of the BrainSpan-derived developmental signature score in the GSE30272 cohort. Supplementary Figure S3. Connectivity distribution and functional robustness of the BrainSpan-derived primary developmental module. (A) Distribution of absolute module membership (|kME|) across genes assigned to the primary developmental module. Dashed vertical lines indicate the prespecified |kME| thresholds of 0.75 and 0.85 used to define progressively higher-confidence module representations for cross-dataset transfer and sensitivity analyses. (B) Functional enrichment profiles of the full primary module and the higher-confidence |kME| ≥ 0.75 and |kME| ≥ 0.85 subsets. Representative enriched biological processes are shown across the three module definitions. Dot size represents enrichment significance as −log10(FDR). The persistence of major developmental, neuronal, RNA-processing, and translational categories across progressively more stringent kME thresholds indicates that the functional interpretation of the primary module is not driven solely by lower-membership genes. Supplementary Figure S4. Sensitivity analysis of module-membership estimates after covariate adjustment. (A) Relationship between original WGCNA-derived module membership and module membership recalculated after residualization of gene expression for developmental age and major sample-level covariates within the predefined turquoise-module gene set. Module-membership estimates showed moderate concordance after adjustment (Spearman ρ = 0.418). (B) Overlap of highly ranked genes before and after residualization. Bars show the fraction of genes shared between the original and adjusted top 50, top 100, and top 200 module-membership rankings. The limited overlap indicates substantial reorganization of hub ranking after covariate adjustment. Because the original module membership was retained rather than a genome-wide residual network being reconstructed, these analyses assess internal concordance only and do not constitute a formal test of module or network preservation; Table S1: Complete membership of the BrainSpan-derived postnatal-rising turquoise primary developmental module. Table S2: Higher-confidence transferable signature derived from the BrainSpan turquoise primary developmental module. Table S3: BrainSpan turquoise primary developmental module. Table S4: Human-to-mouse ortholog mapping for the corrected BrainSpan turquoise transferable signature. Table S5: Cross-species mapping retention summary and scoring definition for the corrected BrainSpan turquoise transferable signature. Table S6: WGCNA module sizes, canonical clock-gene enrichment, and module membership statistics across BrainSpan modules. Table S7: Human BTS atlas composition and corrected turquoise-signature projection summary. Table S8: Sample and developmental-stage composition and processing summary for the GSE153162 wild-type mouse cortical single-cell dataset. Table S9: Litter-level design summary for the GSE166376 maternal immune activation dataset.
