# Supplementary material for: Clock-Related Genes Mark a Developmental Cortical Maturation Program Associated with Stage-Resolved Responses to Prenatal Immune Activation
Source: Genes (Basel). 2026 Sep 12;17(9):1107. doi: 10.3390/genes17091107 (PMC13606453; doi:10.3390/genes17091107)

## Supplementary Figure S1

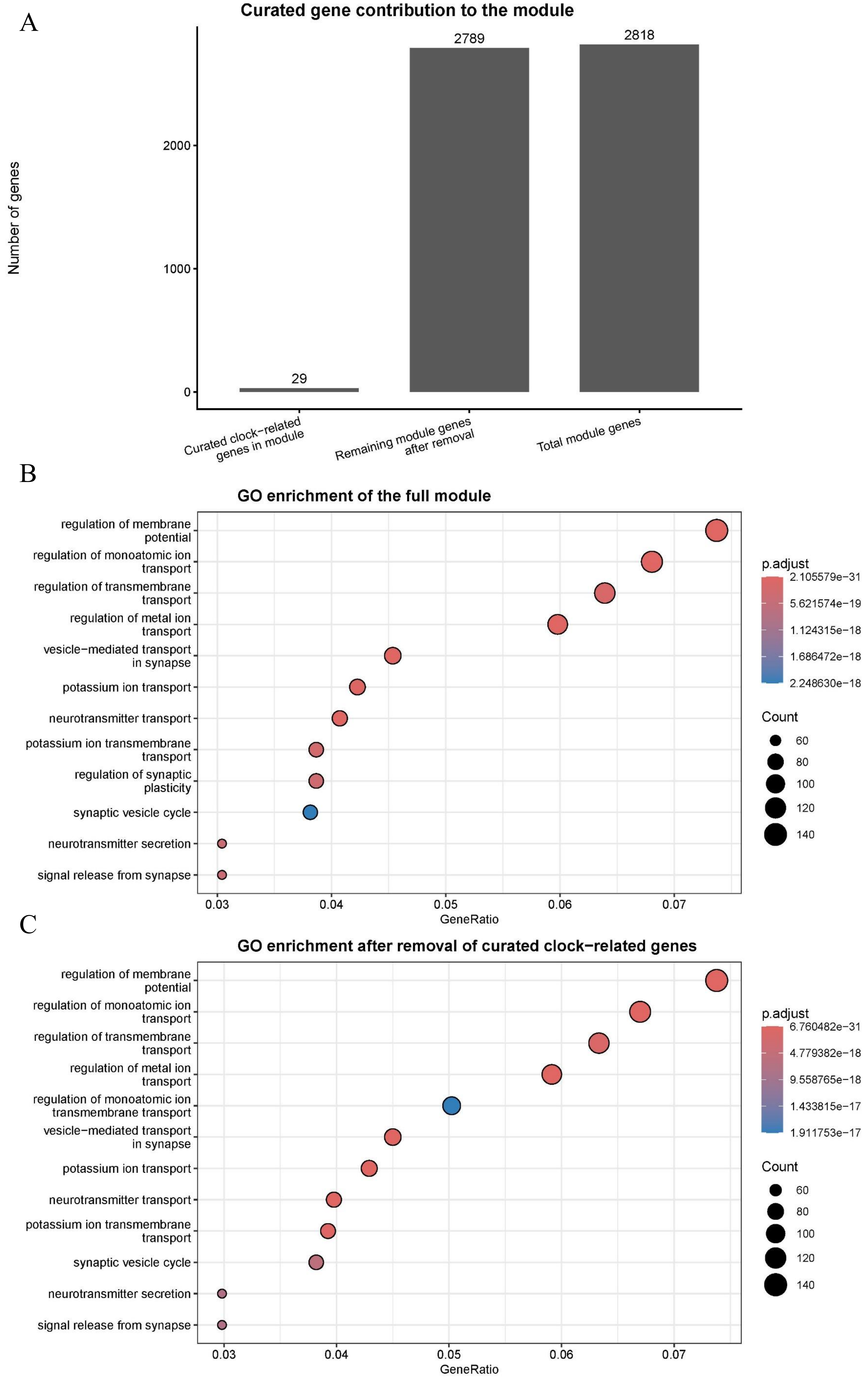

# Supplementary Figure S2

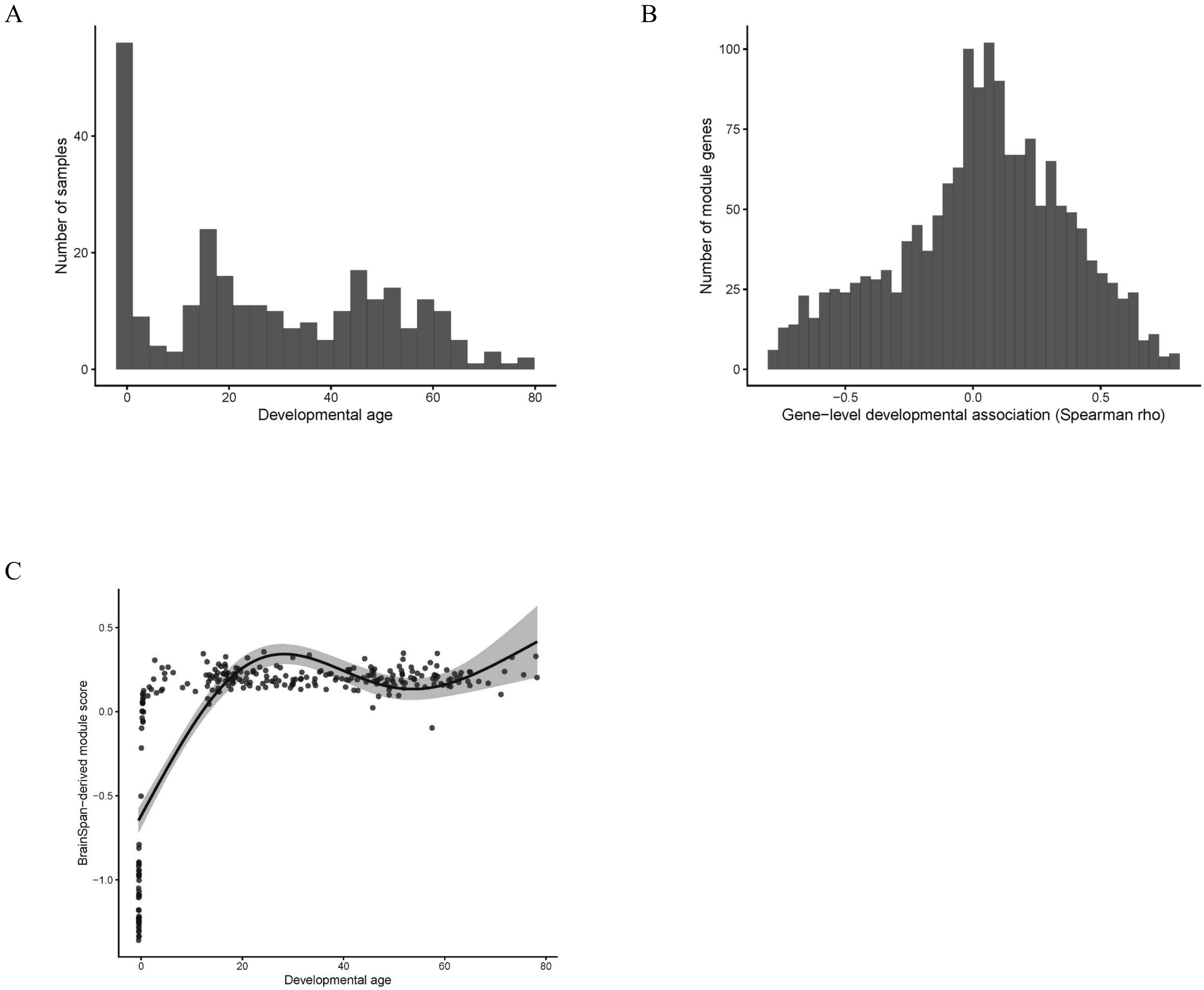

# Supplementary Figure S3

A

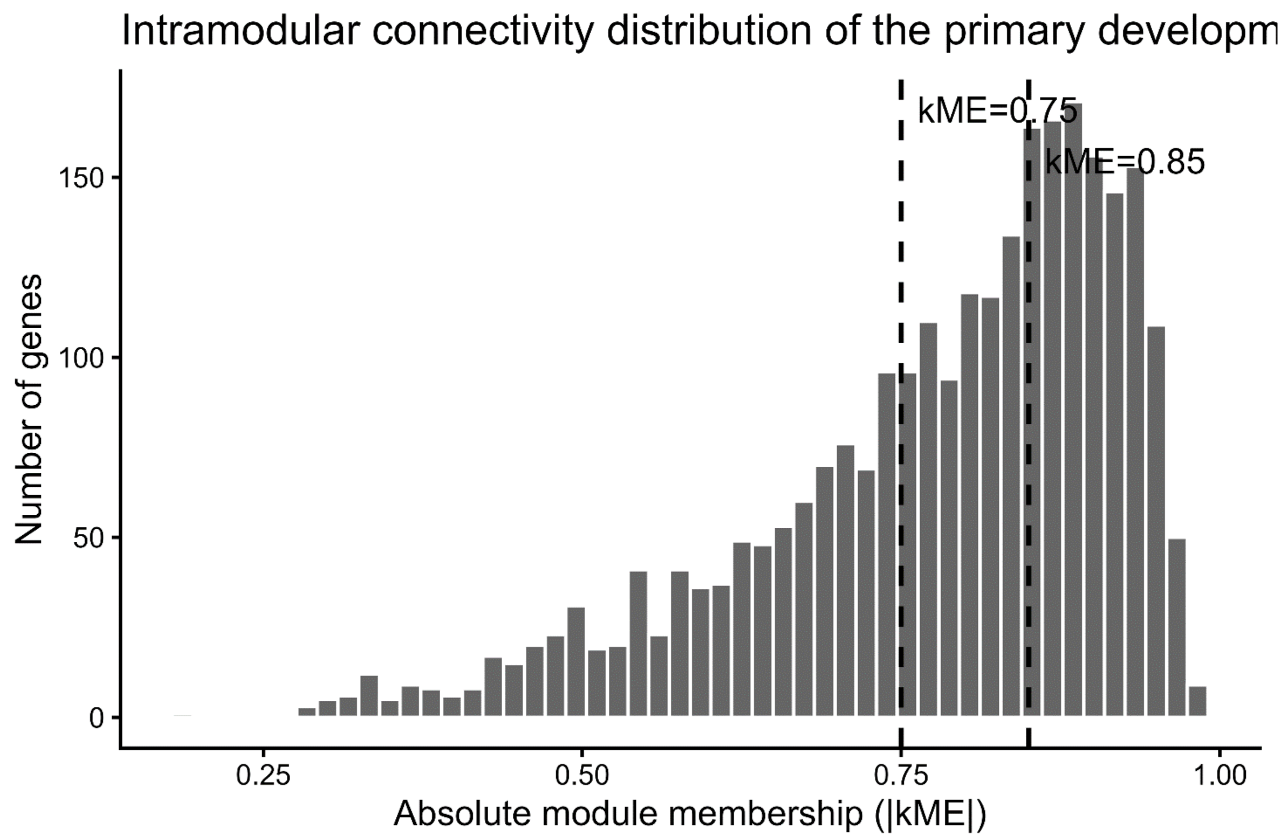


B

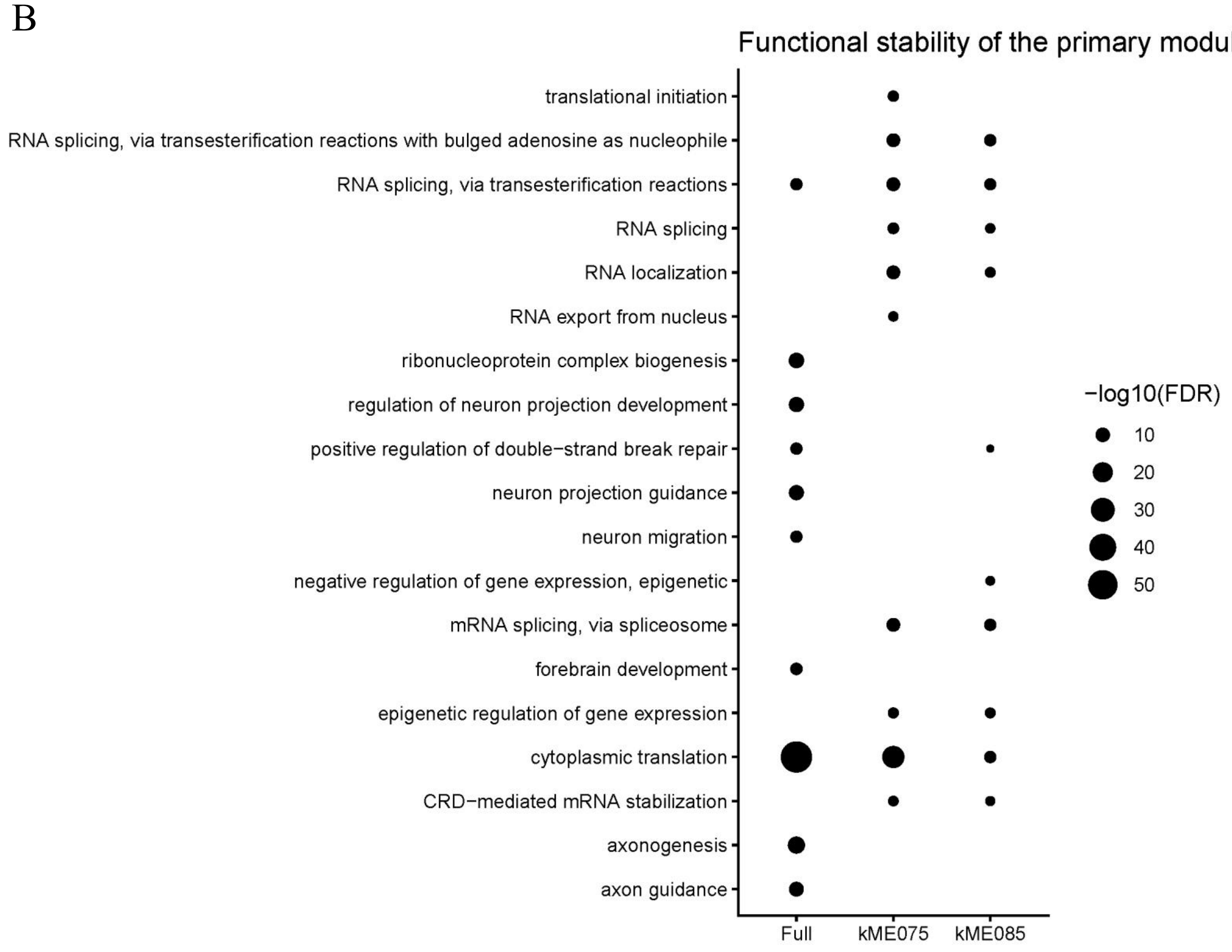

# Supplementary Figure S4

A

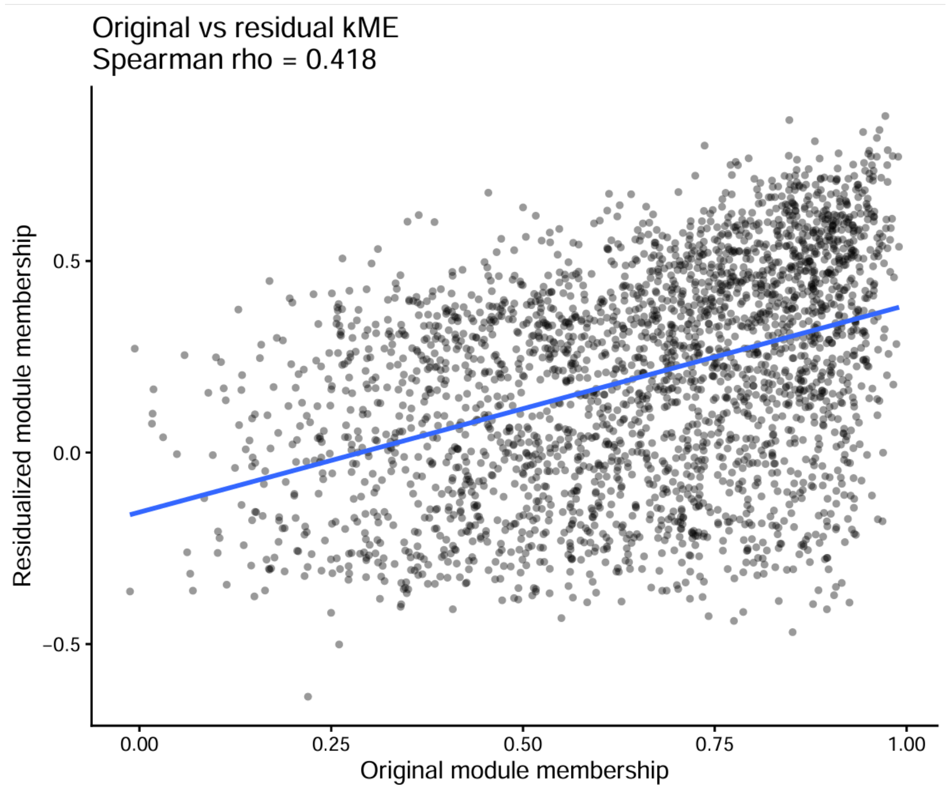


B

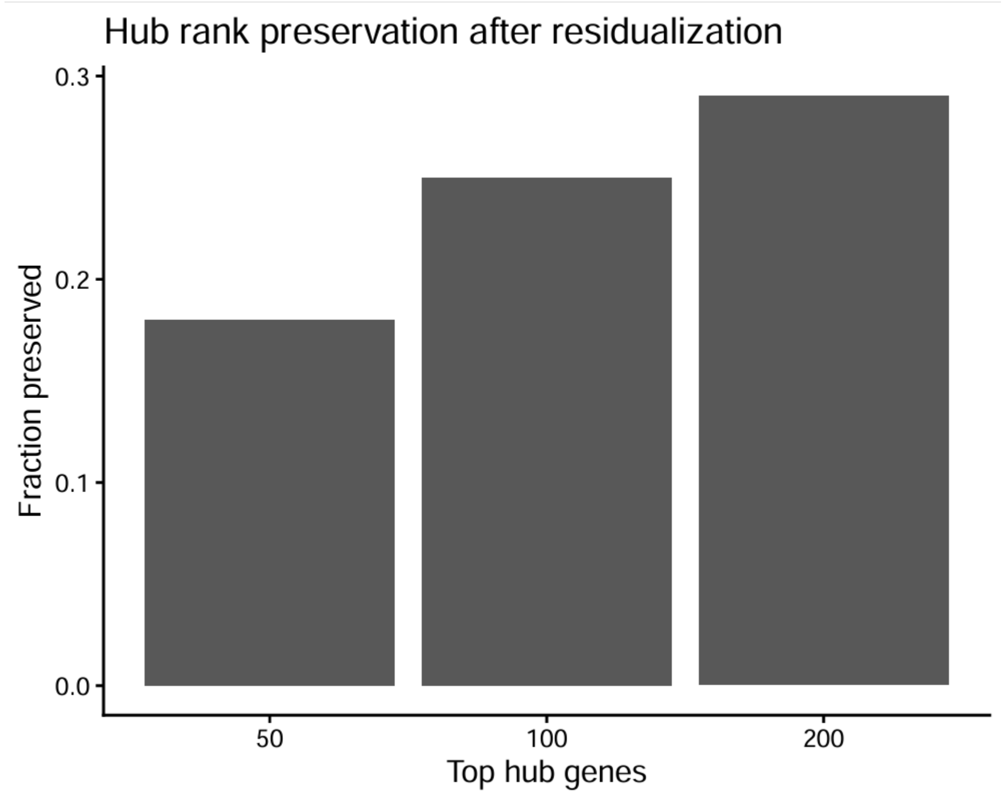

Supplement: Supplementary file 1 [file genes-17-01107-s001.zip › Sup Figure-genes-R2.pdf]
